# Gene expression anti-profiles as a basis for accurate universal cancer signatures

**DOI:** 10.1186/1471-2105-13-272

**Published:** 2012-10-22

**Authors:** Héctor Corrada Bravo, Vasyl Pihur, Matthew McCall, Rafael A Irizarry, Jeffrey T Leek

**Affiliations:** 1 Department of Computer Science, Center for Bioinformatics and Computational Biology, University of Maryland, College Park, MD, USA; 2 Department of Biostatistics, Johns Hopkins Bloomberg School of Public Health, Baltimore, MD, USA; 3 Department of Biostatistics and Computational Biology, University of Rochester Medical Center, Rochester, NY, USA

**Keywords:** Gene expression, Cancer, Genomic signatures, Microarray normalization and quality assessment, Anti-profiles

## Abstract

**Background:**

Early screening for cancer is arguably one of the greatest public health advances over the last fifty years. However, many cancer screening tests are invasive (digital rectal exams), expensive (mammograms, imaging) or both (colonoscopies). This has spurred growing interest in developing genomic signatures that can be used for cancer diagnosis and prognosis. However, progress has been slowed by heterogeneity in cancer profiles and the lack of effective computational prediction tools for this type of data.

**Results:**

We developed *anti-profiles* as a first step towards translating experimental findings suggesting that stochastic across-sample hyper-variability in the expression of specific genes is a stable and general property of cancer into predictive and diagnostic signatures. Using single-chip microarray normalization and quality assessment methods, we developed an *anti-profile* for colon cancer in tissue biopsy samples. To demonstrate the translational potential of our findings, we applied the signature developed in the tissue samples, without any further retraining or normalization, to screen patients for colon cancer based on genomic measurements from peripheral blood in an independent study (AUC of 0.89). This method achieved higher accuracy than the signature underlying commercially available peripheral blood screening tests for colon cancer (AUC of 0.81). We also confirmed the existence of hyper-variable genes across a range of cancer types and found that a significant proportion of tissue-specific genes are hyper-variable in cancer. Based on these observations, we developed a universal cancer *anti-profile* that accurately distinguishes cancer from normal regardless of tissue type (ten-fold cross-validation AUC > 0.92).

**Conclusions:**

We have introduced *anti-profiles* as a new approach for developing cancer genomic signatures that specifically takes advantage of gene expression heterogeneity. We have demonstrated that *anti-profiles* can be successfully applied to develop peripheral-blood based diagnostics for cancer and used *anti-profiles* to develop a highly accurate universal cancer signature. By using single-chip normalization and quality assessment methods, no further retraining of signatures developed by the anti-profile approach would be required before their application in clinical settings. Our results suggest that *anti-profiles* may be used to develop inexpensive and non-invasive universal cancer screening tests.

## Background

Early detection through mass screening remains one of the most effective approaches for reducing health care costs [1-4] and mortality [5-10] due to cancer. Despite the benefits, there remain significant barriers to cancer screening including cost [11,12], lack of insurance [11,13], and anxiety or embarrassment about invasive procedures [11,12,14]. There are also cancer types for which mass-screening tools have not been developed [15,16]. Reducing the cost and inconvenience of screening may lead to increased early screening and potentially improve patient and health economic outcomes.

Peripheral blood-based genomic signatures are a promising avenue for developing non-invasive cancer biomarkers [17-21]. However, lack of stable markers in cancer gene expression profiles and associated blood samples has made finding robust screening biomarkers difficult. Here we take advantage of a new theoretical model for evolutionary fitness that suggests that a defining characteristic of cancer is increased epigenetic and gene expression variability [22]. Supporting evidence was provided by the observation of increased variability in DNA methylation across five different cancer types [23]. This model implies that a stable characteristic is that certain genes will consistently show higher across-sample variability in cancer as compared to normal samples. We present a statistical technique that leverages this characteristic by identifying genes that show normal variation in healthy samples, but hyper-variability across tumor samples and use these genes to predict outcome using what we refer to as an *anti-profile*. We define an *anti-profile* score for a specific sample as the number of hyper-variable genes for which expression in that sample falls outside a defined range of normal expression (see Methods for details). We illustrate the technique on a colon cancer dataset, suggest its potential by predicting cancer in a peripheral blood dataset, and explore the possibility of a universal cancer predictor by simultaneously predicting outcome with data from 52 cancer types. All datasets were obtained from public repositories.

We complement our novel statistical approach with new biological insights related to cancer. For the colon cancer anti-profiles we incorporate the finding that consistent decreases in methylation are observed along large (5kb – 10Mb) genomic blocks [23]. Specifically, we only considered genes that lie inside these blocks for the colon cancer anti-profile. For the universal anti-profile we incorporated the finding that genes showing epigenetic hyper-variability in cancer tend to be tissue specific genes [23-25]. We therefore restricted genes in our universal cancer anti-profile to tissue-specific genes.

Gene expression variability and stochasticity have been studied previously in the context of normal populations [26,27], with recent work exploring the role of genetic variants in altering expression variation and stochasticity [28]. Of particular interest is recent work showing a link between variation in normal populations and HIV susceptibility [29]. It is only recently, however, that direct association between gene expression variability and disease has been studied on neurological disease [23,30] and cancer [23]. We show that increased variability in specific genes is a characteristic feature in many cancer types that can be used for prediction. The *anti-profile* method we propose here is an application to the predictive setting of ideas in existing statistical methods developed to identify and model outliers in gene expression due to cancer [31,32]. Here we expand these ideas and leverage our knowledge of and experience with preprocessing and normalization of high-throughput expression data to describe and demonstrate the effectiveness of the anti-profile method to develop signatures based on technology ready to be used in clinical settings (through quality assessment and normalization) and a general and stable cancer marker (increased gene expression hypervariability of specific genes).

## Results and discussion

### Gene expression anti-profiles

We developed the *anti-profile* method as a simple and robust approach to define cancer genomic signatures by specifically taking advantage of heterogeneity in cancer. An important first step in our approach is to normalize raw gene expression data; an often-overlooked, but key issue in the development of genomic signatures based on microarray data. Standard microarray normalization methods cannot be used when developing clinical diagnostics since they require multiple samples and normalized values depend on which samples are normalized together [33,34]. This means that signatures can only be translated to the clinic after independent retraining of the signatures is performed with single-sample normalization techniques [35]. For all signatures developed here, we employ a recently developed single-sample normalization technique for microarrays [36] and a single-array quality metric [37]. Since signatures are developed with single-sample normalization, they can be directly used as clinical diagnostics, without further retraining.

**Figure 1:**
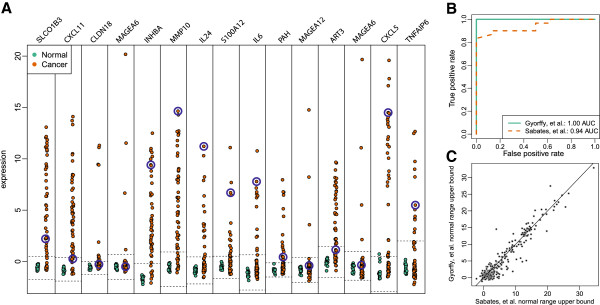
**The colon cancer anti-profile signature.** (**A**) Normalized gene expression for 15 hyper-variable genes in cancer from two independent colon cancer datasets [38-40]. Normal samples are shown in green, cancer samples are shown in orange. We define the anti-profile as the set of genes and a corresponding range of normal expression values for each gene (indicated by dotted lines). Only genes inside colon methylation blocks [23] were included. The *anti-profile* score for each sample is the number of genes in the signature that are outside their defined range of expression. Blue circles highlight expression for one specific cancer sample with an anti-profile score of 9. (**B**) ROC curves using the anti-profile method trained on one colon cancer study to score samples from an independent colon cancer study. The anti-profile includes genes inside colon DNA methylation change blocks where across-sample variance in cancer is at least twice that of normal in the training study. The anti-profile method is very accurate (ROCs of 0.94 and 1.00). (**C**) We compare the upper bounds of normal expression (median + 5*median absolute deviation) as defined by the two independent colon cancer studies and find that ranges are highly consistent.

To illustrate our method we developed an expression anti-profile that distinguishes colon cancer from normal colon in tissue biopsies. We used two independent colon cancer studies, performed by different groups [38-40], as an example. We designated one of these datasets as a training set [38,39] and looked at genes inside reported colon methylation change blocks [23] to select those that showed hyper-variability within colon cancer samples compared to normals. This dataset [38,39] includes premalignant lesions (adenomas) which we treated as a separate biological class and were not included in the following analysis. We applied the resulting anti-profile signature on the independent testing colon cancer dataset in biopsies [40] to evaluate its accuracy and observed area under the ROC curve (AUC) of 0.94 (Figure 1B) with 76% accuracy. We also performed the same experiment with training and testing sets reversed and obtained an AUC of 1.0 with 86% accuracy. We found that the normal ranges of expression defined independently by the two colon cancer experiments were stable (Figure 1C), consistent with the observation that these genes are tightly regulated in normal tissue.

To determine the relationship between gene expression hyper-variability and CpG DNA methylation hyper-variability, we examined a publicly available DNA methylation dataset comparing colon cancer with matched normal colon tissue on the Illumina HumanMethylation 27k BeadChip array (see Methods). We found that there is significant overlap between genes with hyper-variable expression in colon cancer and promoter region CpG hyper-variable methylation (Fisher’s exact test OR=2.41, P=0.005, see Methods). We then repeated the experiment on the two colon cancer expression datasets using CpG hyper-variable methylation to select anti-profile genes and observed worse prediction performance (AUC=.84 and AUC=.97). Enrichment of hyper-variable CpG DNA methylation in blocks of hypo-methylation for this dataset has been previously reported [23]. Considering the reduced coverage of the 27k array, which is biased towards CpG islands, this prediction result indicates the advantage of using hypo-methylation blocks in cancer as a stable and comprehensive proxy for methylation hyper-variability in the absence of suitable direct measurements.

### Colon cancer biomarker in peripheral blood

**Figure 2:**
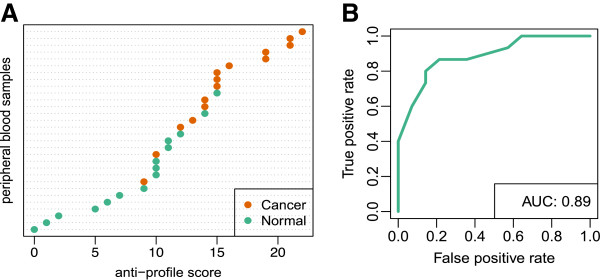
**The colon cancer peripheral blood anti-profile.** (**A**) Plot of the *anti-profile* scores calculated with the colon tissue anti-profile on an independent peripheral blood study without retraining [19]. (**B**) ROC curve and AUC value for the *anti-profile* prediction on the independent peripheral blood study. The *anti-profile* method achieves an AUC of 0.89 without any retraining.

We combined the two colon-cancer tissue datasets described above and derived one *anti-profile* signature (542 genes). We directly applied the anti-profile derived from colon tissue to publicly available peripheral blood samples that passed quality assessment (see Methods section for details) from cancer patients (n=15) and normal samples (n=15) without any retraining [19]. We were able to accurately identify colon cancer samples from peripheral blood (AUC 0.89, Figure 2 and Additional file 1: Figure S1). Without retraining, the accuracy of our *anti-profile* signature was equivalent to the training-set accuracy achieved by the 5-gene score developed by Han et al. [19] directly on these blood samples (AUC =0.88). Estimated training-set accuracy is known to be an overestimate of the true out of sample accuracy for a signature [41], so we also tested the five-gene signature using logistic regression and found its leave-one-out AUC to be 0.81 (P-value=0.19 for test of differences between this and the AUC for the anti-profile signature). We note that further optimization of our *anti-profile* for this task is possible by selecting the optimal number of genes based on performance on the peripheral blood samples themselves. For instance, a slightly larger *anti-profile* signature (650 genes) achieved an AUC of 0.93 (Additional file 1: Figure S1, P-value=0.08 for test of differences between AUCs). However, this type of optimization should be based on datasets with more samples than available here and thus we didn’t pursue this avenue further.

### Consistent hyper-variability across cancer types

We collected and manually curated a set of 6,172 cancer and normal microarray samples in biopsies (n=4,950 and n=1,222 respectively) from 59 tumor types and 102 normal tissue types across 176 different studies in the Gene Expression Omnibus (GEO, [42]). Additional file 1: Table S1 lists the GEO accession number of experiments included in the dataset after removing samples that did not pass the single-chip quality filtering criteria, along with the tissue or tumor type and clinical characteristics annotated in each experiment. These data represent all the clinical information available about each of these samples in GEO. For each tissue or tumor type the number of biological replicates varied and for seven tissue types (adrenal cortex, colon, endometrium, kidney, skin, stomach and vulva) we had at least 10 samples of each of normal tissue and corresponding tumor type.

**Figure 3:**
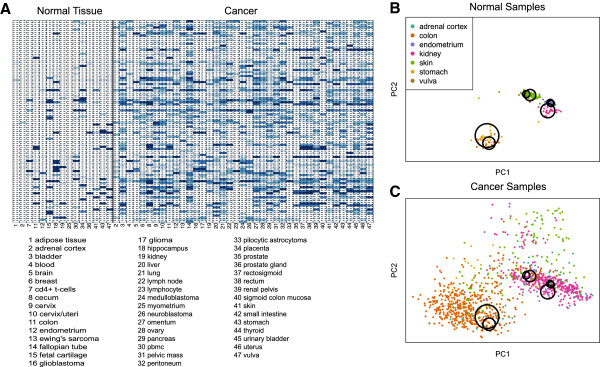
**Genes with consistent hyper-variability across cancer types.** (**A**) The 100 genes that most consistently show hyper variability across cancer types. We first define a normal range of expression using normal samples across all tissue types expecting that normal samples from a few tissue types will deviate from this normal range due to the tissue specificity of some genes. Each cell in the matrix indicates the percentage of samples of each type in which expression is outside the normal range. We observed that for the majority of genes, the percentage of samples in each normal tissue type outside normal range is close to either 0% (most tissues) or 100% (the small number of tissues for which the gene is specific). We also observed that in cancer, percentages are consistently away from 0% or 100%, indicating high variability. (**B**) Principal components for normal samples in adrenal cortex, colon, endometrium, kidney, skin, stomach and vulva. Circles illustrate profiles of normal expression for each tissue type. (**C**) Principal components for cancer samples. Increased variability is present in cancer but not manifested as multiple tightly defined sub-groups for each cancer type. Instead, we observe lack of regulation in cancer around tightly regulated regions of normal expression in each tissue type. The anti-profile method is based on this observation: stochastic departure from tightly regulated normal expression in these genes is characteristic in cancer and can be used in predictive settings.

Using these data we developed an anti-profile to predict cancer status regardless of tumor or tissue type. First, we confirmed that across-sample variability was a general characteristic of cancer (Additional file 1: Figure S2). We selected hyper-variable genes and defined normal ranges as described above (details on the few technical differences are described in the Methods section). Looking at the top 100 genes that showed consistent hyper-variability in cancer we found they were consistently unexpressed in most normal tissues while consistently expressed in a few normal tissues (Figure 3A). In contrast, no consistency of expression was observed in cancer (Figure 3A). We observed the same pattern on an independent set of samples not used to define hyper-variable genes (Additional file 1: Figure S3). We confirmed that hyper-variable genes in cancer coincide with tissue specific genes (Figure 3B and C, Additional file 1: Figure S4). Specifically, we found that the set of tissue-specific genes were enriched for universally hyper-variable genes (Fisher test, odds-ratio 3.1, P<2.2e-16, Additional file 1: Figure S5). Gene ontology category enrichment analysis [43] performed on the anti-profile genes found that categories involving development, organ morphogenesis and differentiation are enriched with hyper-variable genes (Additional file 1: Table S2).

### Consistent hyper-variability across cancer is not due to cellular heterogeneity

Our results suggest that the universally consistent gene expression hyper-variability we report here cannot be fully ascribed to cellular heterogeneity in cancer samples. For a gene to show hyper-variability in cancer due to cellular heterogeneity, it must also be a marker for a number of distinct cell types in a heterogeneous cellular mixture found in a tumor. However, we found that a large number (45%) of universally hyper-variable genes in cancer are not consistently expressed in any of the normal tissues in our dataset (we say a gene is consistently expressed for a tissue if it is expressed in at least 95% of the normal samples for that tissue, see Methods section). This implies that, for almost half of the universally hyper-variable genes in cancer, hyper-variability cannot be the result of a heterogeneous mixture of markers for different cellular subtypes since these genes are usually silenced in normal tissues. Also, while hyper-variable genes are enriched in the set of tissue-specific genes, we found that the majority of tissue-specific genes are not consistently hyper-variable (64%). The vast majority of tissue-specific genes show hyper-variability in a small number of cancer types (Additional file 1: Figure S6) as expected from a histologically heterogeneous sample. This suggests that the lack of regulation of the particular tissue-specific genes that are consistently hyper-variable across cancer types represents a specific and general characteristic of cancer.

We also investigated the relationship between cancer-specific hyper-variability and tissue-specificity in the seven tissues for which we have sufficient samples of both normal and cancer. We found that the vast majority (95-99%) of hyper-variable genes in each of these cancers are not tissue-specific for the corresponding normal tissue (Additional file 1: Table S5). However, hyper-variable genes in each of these cancers are enriched in the set of genes that are specific for the corresponding normal tissue, although the number of genes is small. This small set of genes could indeed include those where hyper-variability in that specific cancer is due to cellular heterogeneity, as normal cells may be included in varying proportions in these tumor samples. We looked at the relationship between cancer-specific differential expression, determined using Empirical Bayes methods [44] as fold-change greater than 1 and significance less than 10% FDR, and tissue-specificity in the same seven tissues. Similar to hyper-variability we found that the vast majority of differentially expressed genes in each of these cancers are not tissue-specific for the corresponding normal tissue. However, in contrast to hyper-variable genes there is no enrichment of differentially expressed genes in the set of genes that are specific for the corresponding normal tissue.

Considering this finding, we investigated the relationship between cellular-specificity and the colon cancer peripheral blood result reported above. We determined genes that are specific to strictly one of two types of lymphocytes for which we had five or more samples in our dataset (CD4+ and CD31+ T-cells) and found that 12% of the genes used in the peripheral blood colon cancer anti-profile fall under this category. Furthermore, lymphocyte-specific genes are enriched in the set of genes with hyper-variable expression in colon cancer inside colon cancer hypo-methylation blocks (Fisher’s exact test OR 3.0, P=1.2e-11). This suggests that we cannot rule out that varying lymphocyte composition in the peripheral blood samples of colon cancer patients may drive the prediction performance of the peripheral blood anti-profile.

### Universal cancer anti-profile

**Figure 4:**
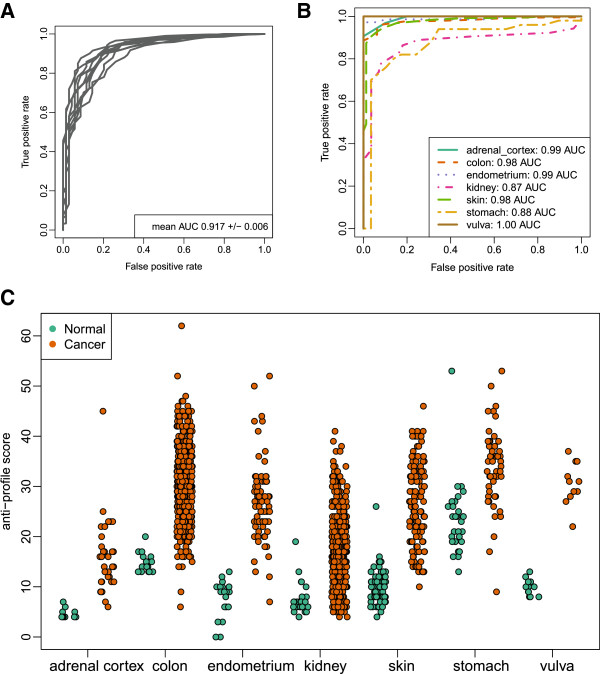
**A stochastic universal cancer classifier.** (**A**) ROC curves for a 10-fold cross-validation experiment classifying *any* sample as normal or tumor, where the anti-profile is trained (genes selected and normal regions of expression defined) independently for each fold, and the ROC is computed for each testing fold independently. (**B**) ROC curves for 7 leave-one-tissue-out experiments. In each of the leave-one-tissue-out experiments, all samples of that particular type (both normal and tumor) are removed from training sets and then scored using the resulting anti-profiles. (**C**) Cross-validated anti-profile scores for the 7 leave-one-tissue-out experiments. The anti-profile scores can separate a large number of tumors from their corresponding normal samples.

While in the colon cancer anti-profile we restricted genes to be in the colon-cancer hypo-methylated blocks here we used our newly found biological insight: we restricted the anti-profile to tissue-specific genes defined as those genes that are expressed in at least 95% of samples for at most three tissues using the gene expression barcode method [45]. With an anti-profile classification in place, we then quantified the accuracy of this universal anti-profile method by performing two cross-validation experiments. We first performed a 10-fold cross validation experiment where an anti-profile was constructed on the training set of each cross-validation fold. The procedure was highly accurate with an average area under the ROC curve (AUC) across the 10 cross-validation experiments of 0.92 (Figure 4A). We next performed a novel leave-one-tissue out cross-validation experiment. For each of the seven tissues for which we had both normal and cancer samples, we defined an anti-profile using samples from the other six tissues and scored samples from the tissue being tested (Figure 4B and C). For all experiments, the leave-one-tissue-out anti-profiles achieved AUCs greater than 0.87. We also observed that the set of probes consistently selected across cross-validation experiments is very stable, indicating the robustness of the anti-profile procedure (Additional file 1: Figure S7). Our analysis indicates that the anti-profile method is able to accurately distinguish tumors from normal samples on tissues not included in its training set and further suggests the universal applicability of the anti-profile method.

We used pathological tumor stage or grade annotation available for a subset of the samples used in the leave-one-tissue-out cross-validation experiment to determine if heterogeneity across samples in pathological tumor stage or grade may explain the increased gene expression variability observed in anti-profile genes used for prediction. For each of the leave-one-tissue-out experiments reported in Figure 4, we used an F-test to find genes that are differentially expressed across pathological stages or grades (FDR<0.1, Additional file 1: Table S6). We then applied a Fisher exact test to determine if the 100-gene anti-profile signature used in the leave-one-out-tissue experiment overlapped this set of differentially expressed genes. We found very few genes that are differentially expressed across pathological tumor stage or grade for adrenal cortex, stomach and vulva (22, 2 and 4 respectively). For the remaining experiments no substantial overlap was observed (OR<2, P-value<0.05). This suggests that increased gene expression variability in anti-profile genes is not explained by heterogeneity of pathological tumor stage or grade in our samples.

## Conclusions

We have introduced and developed gene expression anti-profiles for cancer biomarker discovery. Anti-profiles explicitly model increased gene expression variability in cancer to define robust and reproducible gene expression signatures capable of accurately distinguishing tumor samples from healthy controls. We have developed an *anti-profile* signature in tissue samples from a colon cancer study and validated our signature in a second independent validation set, collected by a different experimental group. We have also applied this signature directly, without retraining, to classify patients with cancer from normals on the basis of genomic measurements in peripheral blood.

We note that Mammaprint [46,47], one of the most successful genomic cancer biomarkers, fits our notion of an anti-profile: its score is calculated based on the correlation between the test sample and a good prognosis gene expression profile. The failure of other, more complex genomic methods to outperform Mammaprint may be due to their reliance on defining specific cancer profiles [48]. While both Mammaprint and our *anti-profile* method classify samples based on deviation from a reference profile, there are two significant differences in the way Mammaprint and the anti-profile method achieve this: 1) Mammaprint uses tumor samples with good prognosis to determine the reference profile. Since these are tumor samples many of the genes used in the profile may exhibit high variability across the good prognosis group. Defining a stable and robust reference profile is essential to the success of this type of method. 2) Mammaprint uses correlation to measure how samples deviate from the reference profile. Our anti-profile method instead uses a robust measure where deviation is based on the number of the genes for which expression falls outside normal ranges of expression, which are themselves estimated using robust methods. It may be possible to improve on the accuracy of the Mammaprint test by adopting a more robust *anti-profile* based on the methods presented in this paper.

In this case we can use the anti-profile score, that is, the number of genes in the anti-profile where expression deviates from a normal range of expression obtained from normal breast tissue samples, to determine prognosis. Since this score is based on stable expression in normal tissues, it may be more robust than calculating correlation to a mean signature for tumors with good prognosis that would show high variability. This will require that more samples of both normal breast tissue and tumor are available on platforms for which robust, single-chip normalization methods exist.

In addition to developing a peripheral blood signature for colon cancer, we have confirmed the existence of hyper-variable genes across 59 distinct cancer types. We also provide evidence of the close relationship between hyper-variability across cancer types and tissue-specific gene expression. Consistent with these observations on tissue-specificity, gene ontology category enrichment analysis found that categories involving development, organ morphogenesis and differentiation are enriched with hyper-variable genes and the remaining gene categories enriched with hyper-variable genes involved cellular interaction with extracellular matrix, e.g., adhesion, localization and collagen catabolic processing or in cell locomotion and cellular component movement. These results argue strongly against the observed hyper-variability being a consequence of sample heterogeneity in the cancer samples.

Incorporating this general result on tissue-specificity and hyper-variability we developed anti-profiles able to classify tissue samples across multiple tissue and cancer types, even when a specific cancer/tissue type is not included in the original training set. Our cross-validation results suggest that consistent hyper-variability of a small set of tissue-specific genes is a stable mark of cancer across tissue types. Our results also suggest the potential for developing peripheral blood signatures for cancer diagnostics on the basis of *anti-profiles.*

In the course of achieving these results we have used recently developed statistical preprocessing methods to remove potential artifacts in a way that is applicable to single clinical samples [36]. This is a somewhat unique approach, as genomic signatures are typically derived after applying population-level pre-processing such as RMA or artifact removal such as surrogate variable analysis. That we achieve such high accuracy in public data – known to be subject to a broad range of technical and biological artifacts [37] – speaks to the strength of our methods.

## Methods

### Gene expression Affymetrix microarray data preprocessing

We downloaded CEL files for 6,172 Affymetrix HGU133plus2 microarrays from 176 studies in the Gene Expression Omnibus (GEO, [42]). CEL files were preprocessed with the *frma* ( [36]) single-chip procedure. Expression measurements were standardized using *Gene Expression Barcode z-*scores ( [45]). We removed arrays that were deposited multiple times into the repository (Euclidean distance between arrays less than 1). We used the GNUSE metric ( [37]) to assess array quality and removed all arrays from studies with median GNUSE greater than 1.25 and removed individual arrays with GNUSE greater than 1.2. We did further hand curation to retain only normal tissue and cancer samples (n=688 and n=4,138 respectively). Additional file 1: Table S1 contains the complete list of studies and samples used in the reported analyses including the type of clinical annotation available for each sample. The curated and preprocessed data is available for download at http://cbcb.umd.edu/~hcorrada/antiProfiles.

### Colon cancer anti-profile

We used the HGU133plus2 probeset annotation from Ensembl (version 15, gene dataset version: GRCh37.p5) to map probesets to genes and obtain each gene’s transcription start site. In the colon cancer anti-profile, we only consider probesets for genes with transcription start sites inside blocks of DNA methylation change ( [23], genomic coordinates available at http://www.nature.com/ng/journal/v43/n8/extref/ng.865-S2.xls). We use the ratio of standard deviations across samples as a statistic to select probesets for the anti-profile: *r*_*g*_ = log _2_(*S*_*gc*_/*S*_*gn*_)where s_gc_ is the across-sample standard deviation of expression for probeset g among the colon tumor samples, and s_gn_ is the across-sample standard deviation of expression for probeset g among the normal samples. The anti-profile includes probesets with r_g_>1 (variability in cancer is twice that of normal).

Normal regions of expression are defined for each probeset as median expression +/− 5 median absolute deviations of expression in the normal samples. We found that our results are quite insensitive to the choice of median absolute deviation multiplier (Additional file 1: Figure S8). The anti-profile score for a specific sample is then the number of probesets outside their respective range of normal expression. A cutoff score can be used to turn the anti-profile score into a classification: scores greater than the cutoff are classified as cancer, scores lower than the cutoff are classified as tumor. A specific cutoff can be determined according to a prescribed objective: e.g. maximize accuracy, or maximize specificity at a given sensitivity in a held-aside test set. We used area under the ROC curve [49] to measure anti-profile accuracy and the DeLong method [50] as implemented in the pROC package [51] to test for differences in AUC.

### Colon cancer illumina HumanMethylation 27k array

We downloaded a publicly available dataset of methylation levels of 22 matched colon normal/tumor samples assayed using Illumina’s HumanMethylation 27k array (GEO accession number GSE17648). Methylation measurements were used with no further preprocessing. Differences in methylation variability were determined using an F-test and significance determined at 1% false discovery rate. For each probeset in our expression data we found the CpG inside it’s promoter region (defined as 1000bp upstream and 250bp downstream) nearest to the transcription start site. We determined significant expression hyper-variability using an F-test at 1% false discovery rate to determine overlap between expression hyper-variability and DNA methylation hyper-variability.

### Colon cancer peripheral blood data

We obtained peripheral blood Affymetrix HGU133plus2 samples from colon cancer patients and healthy controls ( [19] from the study authors, and [52] from GEO with accession number GSE10715). Arrays were preprocessed with fRMA and normalized using the gene expression barcode. Arrays with GNUSE values >1.2 were removed, which left 15 colon cancer samples and 15 normal samples from the first study. Median GNUSE for the second study was 1.46 and thus was not included in the analysis (all but three cancer samples had GNUSE >1.2 in this study).

### Colon cancer peripheral blood anti-profile signature

We defined the anti-profile from colon tissue by combining samples from the two colon cancer biopsy datasets used in the *Gene Expression Antiprofiles* Results section [38,40,52]. Probesets were included in the anti-profile and regions of normal expression defined as described above. No retraining was done to test on the blood dataset. The list of genes and corresponding median and median absolute deviation of expression are given in Additional file 2: Table S3.

To assess the sensitivity to signature size of the accuracy of the peripheral blood signature, we tested signatures of increasing size with genes included in order of decreasing hyper-variability across colon tumor samples (Additional file 1: Figure S1). While the signature reported in the manuscript obtained an AUC of 0.89, similar AUCs are obtained with signatures with about 500–2000 genes inside blocks indicating that the prediction result reported in the manuscript is not very sensitive to the specific signature size chosen. To ascertain significance of the prediction results obtained we performed a randomization test: for each signature size, we generated 1000 signatures with randomly selected subsets of genes of the appropriate size to build each anti-profile. Ranges of normal expression do not change since these are defined from the colon tissue dataset. We used the proportion of random signatures obtaining an AUC greater than or equal to the anti-profile of the corresponding size as a measure of uncertainty. Results that showed significantly high AUC were signatures that include about 500–2000 of the top hyper-variable genes inside methylation blocks.

### Universal hyper-variable genes in cancer

To determine probesets that exhibit hypervariable expression in cancer we compute a variance ratio statistic across multiple tissues. We restrict this computation to tissues and cancer types with more than 10 samples in our dataset (list given in Figure 3). We compute standard deviation of expression for probeset g (s_gt_) separately for each tissue t and cancer type c (s_gc_)*.* We define the variance ratio statistic u_g_ (Additional file 1: Figure S2) as *u*_*g*_ = log _2_(*mean*_*c*_*s*_*gc*_/*mean*_*t*_*s*_*gt*_).

To define the universal normal range of expression we use a similar method: we compute median expression for each gene g on each tissue t separately (m_gt_) along with median absolute deviation (mad_gt_)*.* The universal range is then defined as m_g_ +/− 5 * mad_g_ where m_g_=median_t_(m_gt_) and mad_g_=median_t_(mad_gt_)*.* The list of hyper-variable genes (u_g_>1) and associated median expression and median absolute deviation of expression are provided in Additional file 3: Table S4.

### Defining tissue-specific genes

To define tissue-specific genes, we tabulated the number of samples in which a gene is expressed (defined as gene expression barcode *z*-score greater than 2.54) for each tissue in our dataset with more than 10 normal samples. Tissue-specific genes were defined as those in which the gene is expressed in more than 95% of the samples of at most three tissues. Fisher’s exact test was used to determine enrichment of hyper-variable genes in the set of tissue-specific genes (Additional file 1: Figure S5).

### Gene ontology category enrichment analysis

Gene ontology (GO) enrichment analysis was done using a hyper-geometric test for association between hyper-variable genes (defined as u_g_>1) and GO terms. We used the implementation in the Bioconductor *GOstats* package ( [43]). We used the q-value ( [53]) method to control for multiple hypothesis testing and report enriched categories with Q<0.05 in Additional file 1: Table S2.

### Cross-validation experiments

We performed two types of cross-validation experiments to quantify the accuracy of universal cancer anti-profiles. The first was ten-fold cross validation, data was randomly split into 10 equal-sized subsets, retaining the proportion of normal and cancer samples from the full dataset in each subset. Each of the 10 subsets (or folds) was used sequentially as a test set, scored using an anti-profile trained on the remaining 90% of the data (this includes all steps: 1) filtering to include only tissue-specific probesets, 2) computing the universal variance ratio u_g_*,* 3) selecting the top 100 genes based on the ratio statistic, and 4) computing the universal normal range of expression).

The other type of cross-validation experiment was carried out on the 7 tissues for which we had at least 10 samples each of normal tissue and tumor. For each tissue type, we performed a leave-one-tissue-out experiment by using all samples (normal and corresponding tumor type) as test set and scored them using an anti-profile trained on the remaining data. This ensures that *no* samples from the corresponding tissue (normal or cancer) are included in the training set. Again, all steps required to train the anti-profile were done completely for each leave-one-tissue-out fold.

To classify a new sample we count the number of anti-profile genes for which their expression fell outside their normal range (Figure 2A). A large number of genes with expression outside the normal range, corresponding to a high *anti-profile* score, are indicative of cancer. To develop a predictor for new samples, a cutoff must be defined on the number of genes outside the normal range. If the anti-profile score is less than the cutoff, the sample is classified as normal, if it is greater than cutoff then the sample is classified as cancer.

## Competing interests

The authors report no competing interests.

## Author’s contributions

HCB, JTL and RAI conceived, designed and performed experiments, analyzed data and drafted the manuscript; VP performed experiments and analyzed data; MM contributed reagents. All authors read and approved the final manuscript.

## Supplementary Material

Additional file 1 **Supplementary Material.** This file contains supplementary Figures and Tables. Click here for file

Additional file 2 **Table S3.** Colon cancer anti-profile. Contains Affymetrix probeset ids and normal expression median and median absolute deviation. Click here for file

Additional file 3 **Table S4.** Universal cancer anti-profile. Contains Affymetrix probeset ids and normal expression median and median absolute deviation. Click here for file

## References

[B1] Vasen HF van Ballegooijen M Buskens E Kleibeuker JK Taal BG Griffioen G Nagengast FM Menko FH Meera Khan P A cost-effectiveness analysis of colorectal screening of hereditary nonpolyposis colorectal carcinoma gene carriers Cancer 1998 82 9 1632 1637 10.1002/(SICI)1097-0142(19980501)82:9<1632::AID-CNCR6>3.0.CO;2-C 9576281

[B2] de Koning HJ van Ineveld BM van Oortmarssen GJ de Haes JC Collette HJ Hendriks JH van der Maas PJ Breast cancer screening and cost-effectiveness; policy alternatives, quality of life considerations and the possible impact of uncertain factors Int J Cancer 1991 49 4 531 537 10.1002/ijc.2910490410 1917154

[B3] Goldie SJ Gaffikin L Goldhaber-Fiebert JD Gordillo-Tobar A Levin C Mahe C Wright TC Cost-effectiveness of cervical-cancer screening in five developing countries N Engl J Med 2005 353 20 2158 2168 10.1056/NEJMsa044278 16291985

[B4] Rulyak SJ Kimmey MB Veenstra DL Brentnall TA Cost-effectiveness of pancreatic cancer screening in familial pancreatic cancer kindreds Gastrointest Endosc 2003 57 1 23 29 12518126 10.1067/mge.2003.28

[B5] Tabar L Fagerberg CJ Gad A Baldetorp L Holmberg LH Grontoft O Ljungquist U Lundstrom B Manson JC Eklund G Reduction in mortality from breast cancer after mass screening with mammography. Randomised trial from the Breast Cancer Screening Working Group of the Swedish National Board of Health and Welfare Lancet 1985 1 8433 829 832 2858707 10.1016/s0140-6736(85)92204-4

[B6] Nystrom L Rutqvist LE Wall S Lindgren A Lindqvist M Ryden S Andersson I Bjurstam N Fagerberg G Frisell J Breast cancer screening with mammography: overview of Swedish randomised trials Lancet 1993 341 8851 973 978 10.1016/0140-6736(93)91067-V 8096941

[B7] Newcomb PA Norfleet RG Storer BE Surawicz TS Marcus PM Screening sigmoidoscopy and colorectal cancer mortality J Natl Cancer Inst 1992 84 20 1572 1575 10.1093/jnci/84.20.1572 1404450

[B8] Andriole GL Crawford ED Grubb RL 3rd Buys SS Chia D Church TR Fouad MN Gelmann EP Kvale PA Reding DJ Mortality results from a randomized prostate-cancer screening trial N Engl J Med 2009 360 13 1310 1319 10.1056/NEJMoa0810696 19297565 PMC2944770

[B9] Mandel JS Bond JH Church TR Snover DC Bradley GM Schuman LM Ederer F Reducing mortality from colorectal cancer by screening for fecal occult blood Minnesota Colon Cancer Control Study. N Engl J Med 1993 328 19 1365 1371 10.1056/NEJM199305133281901 8474513

[B10] Walsh JM Terdiman JP Colorectal cancer screening: scientific review JAMA 2003 289 10 1288 1296 10.1001/jama.289.10.1288 12633191

[B11] Klabunde CN Vernon SW Nadel MR Breen N Seeff LC Brown ML Barriers to colorectal cancer screening: a comparison of reports from primary care physicians and average-risk adults Med Care 2005 43 9 939 944 10.1097/01.mlr.0000173599.67470.ba 16116360

[B12] Lerman C Rimer B Trock B Balshem A Engstrom PF Factors associated with repeat adherence to breast cancer screening Prev Med 1990 19 3 279 290 10.1016/0091-7435(90)90028-I 2377590

[B13] Swan J Breen N Coates RJ Rimer BK Lee NC Progress in cancer screening practices in the United States: results from the 2000 National Health Interview Survey Cancer 2003 97 6 1528 1540 10.1002/cncr.11208 12627518

[B14] Harewood GC Wiersema MJ Melton LJ 3rd A prospective, controlled assessment of factors influencing acceptance of screening colonoscopy Am J Gastroenterol 2002 97 12 3186 3194 10.1111/j.1572-0241.2002.07129.x 12492209

[B15] Furukawa H Diagnostic clues for early pancreatic cancer Jpn J Clin Oncol 2002 32 10 391 392 10.1093/jjco/hyf099 12451033

[B16] Bach PB Silvestri GA Hanger M Jett JR Screening for lung cancer: ACCP evidence-based clinical practice guidelines (2nd edition) Chest 2007 132 3 Suppl 69S 77S 17873161 10.1378/chest.07-1349

[B17] Sheng J Zhang WY Identification biomarkers for cervical cancer in peripheral blood lymphocytes by oligonucleotide microarrays Zhonghua Yi Xue Za Zhi 2010 90 37 2611 2615 21162926

[B18] Aaroe J Lindahl T Dumeaux V Saebo S Tobin D Hagen N Skaane P Lonneborg A Sharma P Borresen-Dale AL Gene expression profiling of peripheral blood cells for early detection of breast cancer Breast Cancer Res 2010 12 1 R7 10.1186/bcr2472 20078854 PMC2880427

[B19] Han M Liew CT Zhang HW Chao S Zheng R Yip KT Song ZY Li HM Geng XP Zhu LX Novel blood-based, five-gene biomarker set for the detection of colorectal cancer Clin Cancer Res 2008 14 2 455 460 10.1158/1078-0432.CCR-07-1801 18203981

[B20] Zander T Hofmann A Staratschek-Jox A Classen S Debey-Pascher S Maisel D Ansen S Hahn M Beyer M Thomas RK Blood-based gene expression signatures in non-small cell lung cancer Clin Cancer Res 2011 17 10 3360 3367 10.1158/1078-0432.CCR-10-0533 21558400

[B21] Osman I Bajorin DF Sun TT Zhong H Douglas D Scattergood J Zheng R Han M Marshall KW Liew CC Novel blood biomarkers of human urinary bladder cancer Clin Cancer Res 2006 12 11 Pt 1 3374 3380 16740760 10.1158/1078-0432.CCR-05-2081

[B22] Feinberg AP Irizarry RA Evolution in health and medicine Sackler colloquium: Stochastic epigenetic variation as a driving force of development, evolutionary adaptation, and disease Proc Natl Acad Sci USA 2010 107 Suppl 1 1757 1764 20080672 10.1073/pnas.0906183107 PMC2868296

[B23] Hansen KD Timp W Bravo HC Sabunciyan S Langmead B McDonald OG Wen B Wu H Liu Y Diep D Increased methylation variation in epigenetic domains across cancer types Nat Genet 2011 43 8 768 775 10.1038/ng.865 21706001 PMC3145050

[B24] Irizarry RA Ladd-Acosta C Wen B Wu Z Montano C Onyango P Cui H Gabo K Rongione M Webster M The human colon cancer methylome shows similar hypo- and hypermethylation at conserved tissue-specific CpG island shores Nat Genet 2009 41 2 178 186 10.1038/ng.298 19151715 PMC2729128

[B25] Hofmann O Caballero OL Stevenson BJ Chen YT Cohen T Chua R Maher CA Panji S Schaefer U Kruger A Genome-wide analysis of cancer/testis gene expression Proc Natl Acad Sci USA 2008 105 51 20422 20427 10.1073/pnas.0810777105 19088187 PMC2603434

[B26] Storey JD Madeoy J Strout JL Wurfel M Ronald J Akey JM Gene-expression variation within and among human populations Am J Hum Genet 2007 80 3 502 509 10.1086/512017 17273971 PMC1821107

[B27] Stranger BE Nica AC Forrest MS Dimas A Bird CP Beazley C Ingle CE Dunning M Flicek P Koller D Population genomics of human gene expression Nat Genet 2007 39 10 1217 1224 10.1038/ng2142 17873874 PMC2683249

[B28] Jimenez-Gomez JM Corwin JA Joseph B Maloof JN Kliebenstein DJ Genomic Analysis of QTLs and Genes Altering Natural Variation in Stochastic Noise PLoS Genet 2011 7 9 e1002295 10.1371/journal.pgen.1002295 21980300 PMC3183082

[B29] Li J Liu Y Kim T Min R Zhang Z Gene expression variability within and between human populations and implications toward disease susceptibility PLoS Comput Biol 2010 6 8 e1000910 10.1371/journal.pcbi.1000910 20865155 PMC2928754

[B30] Mar JC Matigian NA Mackay-Sim A Mellick GD Sue CM Silburn PA McGrath JJ Quackenbush J Wells CA Variance of gene expression identifies altered network constraints in neurological disease PLoS Genet 2011 7 8 e1002207 10.1371/journal.pgen.1002207 21852951 PMC3154954

[B31] MacDonald JW Ghosh D COPA–cancer outlier profile analysis Bioinformatics 2006 22 23 2950 2951 10.1093/bioinformatics/btl433 16895932

[B32] Tibshirani R Hastie T Outlier sums for differential gene expression analysis Biostatistics 2007 8 1 2 8 10.1093/biostatistics/kxl005 16702229

[B33] Irizarry RA Hobbs B Collin F Beazer-Barclay YD Antonellis KJ Scherf U Speed TP Exploration, normalization, and summaries of high density oligonucleotide array probe level data Biostatistics 2003 4 2 249 264 10.1093/biostatistics/4.2.249 12925520

[B34] Li C Wong WH Model-based analysis of oligonucleotide arrays: expression index computation and outlier detection Proc Natl Acad Sci USA 2001 98 1 31 36 10.1073/pnas.98.1.31 11134512 PMC14539

[B35] Glas AM Floore A Delahaye LJ Witteveen AT Pover RC Bakx N Lahti-Domenici JS Bruinsma TJ Warmoes MO Bernards R Converting a breast cancer microarray signature into a high-throughput diagnostic test BMC Genomics 2006 7 278 10.1186/1471-2164-7-278 17074082 PMC1636049

[B36] McCall MN Bolstad BM Irizarry RA Frozen robust multiarray analysis (fRMA) Biostatistics 2010 11 2 242 253 10.1093/biostatistics/kxp059 20097884 PMC2830579

[B37] McCall MN Murakami PN Lukk M Huber W Irizarry RA Assessing affymetrix GeneChip microarray quality BMC Bioinforma 2011 12 137 10.1186/1471-2105-12-137 PMC3097162 21548974

[B38] Gyorffy B Molnar B Lage H Szallasi Z Eklund AC Evaluation of microarray preprocessing algorithms based on concordance with RT-PCR in clinical samples PLoS One 2009 4 5 e5645 10.1371/journal.pone.0005645 19461970 PMC2680989

[B39] Galamb O Spisak S Sipos F Toth K Solymosi N Wichmann B Krenacs T Valcz G Tulassay Z Molnar B Reversal of gene expression changes in the colorectal normal-adenoma pathway by NS398 selective COX2 inhibitor Br J Cancer 2010 102 4 765 773 10.1038/sj.bjc.6605515 20087348 PMC2837560

[B40] Sabates-Bellver J Van der Flier LG de Palo M Cattaneo E Maake C Rehrauer H Laczko E Kurowski MA Bujnicki JM Menigatti M Transcriptome profile of human colorectal adenomas Mol Cancer Res 2007 5 12 1263 1275 10.1158/1541-7786.MCR-07-0267 18171984

[B41] Hastie T Tibshirani R Friedman JH The elements of statistical learning: data mining, inference, and prediction 2009 2 Springer, New York, NY

[B42] Edgar R Domrachev M Lash AE Gene Expression Omnibus: NCBI gene expression and hybridization array data repository Nucleic Acids Res 2002 30 1 207 210 10.1093/nar/30.1.207 11752295 PMC99122

[B43] Falcon S Gentleman R Using GOstats to test gene lists for GO term association Bioinformatics 2007 23 2 257 258 10.1093/bioinformatics/btl567 17098774

[B44] Smyth GK Linear models and empirical bayes methods for assessing differential expression in microarray experiments Stat Appl Genet Mol Biol 2004 3 Pages -, ISSN (Online) 1544-6115 10.2202/1544-6115.1027 16646809

[B45] McCall MN Uppal K Jaffee HA Zilliox MJ Irizarry RA The Gene Expression Barcode: leveraging public data repositories to begin cataloging the human and murine transcriptomes Nucleic Acids Res 2011 39 D1011 D1015 Database issue 10.1093/nar/gkq1259 21177656 PMC3013751

[B46] Van’t Veer LJ Dai H van de Vijver MJ He YD Hart AA Mao M Peterse HL van der Kooy K Marton MJ Witteveen AT Gene expression profiling predicts clinical outcome of breast cancer Nature 2002 415 6871 530 536 10.1038/415530a 11823860

[B47] van de Vijver MJ He YD van’t Veer LJ Dai H Hart AA Voskuil DW Schreiber GJ Peterse JL Roberts C Marton MJ A gene-expression signature as a predictor of survival in breast cancer N Engl J Med 2002 347 25 1999 2009 10.1056/NEJMoa021967 12490681

[B48] Koscielny S Why most gene expression signatures of tumors have not been useful in the clinic Sci Transl Med 2010 2 14 14ps2 10.1126/scitranslmed.3000313 20371465

[B49] Fawcett T An introduction to ROC analysis Pattern Recogn Lett 2006 27 8 861 874 10.1016/j.patrec.2005.10.010

[B50] DeLong ER DeLong DM Clarke-Pearson DL Comparing the areas under two or more correlated receiver operating characteristic curves: a nonparametric approach Biometrics 1988 44 3 837 845 10.2307/2531595 3203132

[B51] Robin X Turck N Hainard A Tiberti N Lisacek F Sanchez JC Muller M pROC: an open-source package for R and S+ to analyze and compare ROC curves BMC Bioinforma 2011 12 77 10.1186/1471-2105-12-77 PMC3068975 21414208

[B52] Galamb O Sipos F Solymosi N Spisak S Krenacs T Toth K Tulassay Z Molnar B Diagnostic mRNA expression patterns of inflamed, benign, and malignant colorectal biopsy specimen and their correlation with peripheral blood results Cancer Epidemiol Biomarkers Prev 2008 17 10 2835 2845 10.1158/1055-9965.EPI-08-0231 18843029

[B53] Storey JD Tibshirani R Statistical significance for genomewide studies Proc Natl Acad Sci USA 2003 100 16 9440 9445 10.1073/pnas.1530509100 12883005 PMC170937

