# Supplementary material for: Gene expression anti-profiles as a basis for accurate universal cancer signatures
Source: BMC Bioinformatics. 2012 Oct 22;13:272. doi: 10.1186/1471-2105-13-272 (PMC3487959; doi:10.1186/1471-2105-13-272)
Supplement: Additional file 1 — Supplementary Material. This file contains supplementary Figures and Tables. [file 1471-2105-13-272-S1.pdf]

Supplementary Material for

Gene expression anti-profiles as a basis for accurate universal cancer diagnostics

Héctor Corrada Bravo[1], Vasyl Pihur[2], Matthew McCall[3], Rafael A. Irizarry[2] and Jeffrey T. Leek[2]

1. Center for Bioinformatics and Computational Biology, Department of Computer Science, University of Maryland, College Park, MD, USA.

2. Department of Biostatistics, Johns Hopkins Bloomberg School of Public Health, Baltimore, MD, USA.

3. Department of Biostatistics and Computational Biology, University of Rochester Medical Center, Rochester, NY, USA.

1. Supplementary Text: Materials and Methods
2. Supplementary Table 1. Curated dataset of Affymetrix HGU133plus2 gene expression microarrays.
3. Supplementary Table 2. Gene ontology category enrichment analysis for hyper-variable genes in cancer.
4. Supplementary Table 5. Anti-profile signatures are not explained by pathological tumor stage or grade heterogeneity
5. Supplementary Figure 1. Stability and significance of colon cancer peripheral blood anti-profile signature.
6. Supplementary Figure 2. Histogram of log2 cancer/normal variance ratio.
7. Supplementary Figure 3. Genes with consistent hyper-variability across cancer types.
8. Supplementary Figure 4. Hyper-variable genes in cancer distinguish normal samples from different brain regions.
9. Supplementary Figure 5. The set of tissue-specific genes are enriched for genes that are hyper-variable in cancer.
10. Supplementary Figure 6. Specificity of the universally hyper-variable tissue-specific genes.
11. Supplementary Figure 7. The anti-profile method produces robust signatures.

Supplementary Table 1. Curated dataset of Affymetrix HGU133plus2 microarrays.

GEO accession number	Number of arrays	Number of normal samples	Number of tumor samples	Tissue/tumor types	Clinical annotation available
GSE10282	43	0	43	skin	biopsy type;M_stage;Braf_status;Nras_status;F_stage
GSE10327	62	0	62	medulloblastoma	Gender;Histology;Metastatic stage
GSE10406	8	8	0	sinus_mucosa	disease state
GSE10445	62	0	62	lung	pT2_status;age;gender;disease state
GSE10696	3	3	0	a431_cells	Organ; Tissue; Disease; Age; Gender
GSE10714	3	3	0	colon	
GSE10791	1	1	0	sigmoid_colon	source
GSE10846	154	0	154	lymph_node	Gender;Age;Tissue;Disease state;Individual;Clinical info
GSE10916	48	0	48	skin	treatment;status;BRAF status;NRAS status
GSE10927	64	10	54	adrenal_cortex	age;sex;side of body;Cinical characteristics;tumor diameter (cm);tumor weight (gm);Weiss Grade of tumor;Mitotic rate of tumor;Tumor stage;Adrenocortical Carcinoma cluster in our analysis;Years to last followup;dead or alive at last followup;First principal component for 33 ACCs; Conn's syndrome;
GSE11001	16	0	16	breast	tumor cells;ER;PR;HER2;tumor stage;node stage;grade
GSE11045	3	3	0	kidney	
GSE11151	64	5	59	kidney	
GSE11281	3	3	0	pbmc	
GSE11318	22	0	22	lymph_node	Gender;Age;Tissue;Disease state;Individual;Clinical info
GSE11430;GSE11540	4	4	0	monocytes	Age;Gender;Tissue;Cell type;
GSE11525	3	3	0	pbmc	
GSE11882	42	42	0	hippocampus	Individual;Brain region;Gender;Age
GSE12090	18	0	18	kidney	
GSE12102	36	0	36	ewing's_sarcoma	tumor status
GSE12141	1	0	1	brain	metastasis status
GSE12172	57	0	57	ovary	BRAF status; KRAS statusl Stage; Grade; implant history; metastatic status;
GSE12305	5	5	0	astrocytes;u251_cells	

Supplementary Table 1. Curated dataset of Affymetrix HGU133plus2 microarrays.

GSE12366	3	3	0	memory_b_cells	
GSE12460	28	0	28	neuroblastoma	
GSE12460;GSE14880	28	0	28	neuroblastoma	
GSE12606	4	4	0	kidney	;Gender;Date of birth
GSE12667	71	0	71	lung	Race;Gender;TSP_Patient
GSE12763;GSE12790	29	0	29	breast	tumor type;Her2 status
GSE12767	8	8	0	placenta	race;karyotype status
GSE12875	1	1	0	cd4+_t_cells	
GSE12902	17	0	17	skin	
GSE12992	33	0	33	medulloblastoma	Patient age at diagnosis
GSE13041	27	0	27	glioblastoma	
GSE13067	73	0	73	colon	tumor type
GSE13136;GSE13141	28	0	28	neuroblastoma	Gender;Age;Stage;MYCN CNV status.;1p cnv status;11q cnv status;17q cnv status
GSE13294	127	0	127	colon	tumor type
GSE13351	88	0	88	lymphocyte	subtype
GSE13355	51	51	0	skin	
GSE13471	8	4	4	colon	
GSE13506	44	44	0	adipose_tissue	
GSE13671	8	8	0	breast_epithelial_cells	
GSE13732	20	20	0	cd4+_t_cells	
GSE13738	3	3	0	cd4+_t_cells	
GSE13787	23	0	23	breast	Gender;subtype;Grade
GSE13887	4	4	0	t_cells	
GSE13911	60	23	37	stomach	tumor type
GSE14905	19	19	0	skin	

Supplementary Table 1. Curated dataset of Affymetrix HGU133plus2 microarrays.

GSE2109	1919	0	1919	endometrium;ovary;kidney;colon;uterus;bladder;prostate;breast;omentum;lung;renal_pelvis;thyroid;rectum;rectosigmoid;urinary_bladder;peritoneum;small_intestine;cervix;stomach;liver;esophagus;cervix_uteri;fallopian_tube;brain;pancreas;vulva;axillary_lymph_node;adrenal_gland;lymph_node;small_bowel;parotid_gland;pelvic_mass;sigmoid_colon;corpus_uteri;pelvic_lymph_node;cecum;cervical_lymph_node	Quality metric = 28S to 18S;Patient Age;Gender;Ethnic Background;Tobacco Use ;Alcohol Consumption?;Family History of Cancer?;Days from Patient Diagnosis to Excision;Pathological T;Pathological N;Pathological M;Pathological Stage;Pathological Grade;Pathological Multiple Tumors;Pathological Stage During or Following Multimodality Therapy;Primary Site;Histology;Years of Tobacco Use;Type of Tobacco Use;Presenting Symptoms;Clinical T;Clinical N;Clinical M;Clinical Stage;Clinical Grade;Clinical Multiple Tumors;Clinical Stage During or Following Multimodality Therapy;Diagnosis made by;Pathological Dukes Stage;Pathological Multiple Tumours;Pathological Staged During or Following Multimodality Therapy;PSA;# of years PSA Tested?;Cancer discovered by digital exam?;Clinical Gleason Score;Screening studies performed?;Prior Therapy;Mammogram;Number of years in which a mammogram was obtained?;Oral contraceptives?;Oophorectomy?;Fibrocystic disease?;Mammogram calcifications?;Pathological ER;Pathological PR;Pathological HER/2 Neu;Pathological Metastatic Sites;Workplace or household smokers?;Clinical Multiple Tumours;Clinical Staged During or Following Multimodality Therapy;Relapse Since Primary Treatment;Number of Years Until Relapse;Retreatment T;Retreatment N;Retreatment M;Retreatment Metastatic Sites;Retreatment Stage;Retreatment Grade;Hormonal therapy (;Clinical Question;Clinical Value;Pathological Question;Pathological Value;Have you ever had esophagitis/reflux?;Pathological Gleason Score;Retreatment ER;Retreatment PR;Retreatment HER/2 Neu;Number of years of use?(Oral contraceptives);Clinical Metastatic Sites;Have you ever been diagnosed as having HPV on your PAP smear?;Clinical Dukes Stage;Retreatment Multiple Tumors;Retreatment Stage During or
GSE2677	37	0	37	blood	age
GSE2817	30	0	30	glioma	

Supplementary Table 1. Curated dataset of Affymetrix HGU133plus2 microarrays.

GSE3526	133	133	0	adipose_tissue_omental;bronchus;adipose_tissue;adrenal_gland_cortex;coronary_artery;cervix;cerebellum;heart_atrium;hippocampus;endometrium;kidney_cortex;esophagus;lung;myometrium;mammary_gland;kidney_medulla;lymph_node;pharyngeal_mucosa;ovary;oral_mucosa;stomach_cardiac;saphenous_vein;skeletal_muscle;pituitary;prostate_gland;spleen;stomach_fundus;stomach_pyloric;salivary_gland;tongue_main_corpus;thyroid;vagina;trigeminal_ganglia;tonsil;trachea;vulva;urethra	
GSE3744	47	7	40	breast	
GSE4183	38	8	30	colon	
GSE4237	8	0	8	pituitary	
GSE4780	1	0	1	brain	
GSE5040	4	4	0	gm15851_cells	
GSE5081	5	5	0	stomach	Age;Gender;Tissue
GSE5110	2	2	0	skeletal_muscle	
GSE5460	95	0	95	breast	ER;HER2;B-R grade;node status;LVI;tumor type;tumor size
GSE5563	10	10	0	vulva	Gender
GSE5675	38	0	38	pilocytic_astrocytoma	
GSE5764	4	0	4	breast_lobular_cells	

Supplementary Table 1. Curated dataset of Affymetrix HGU133plus2 microarrays.

GSE5787	33	0	33	cervix	FIGO;Histology;Grade;hp5;HGB;ifp;age;tumsizeMR;ResponseToTherapy
GSE5993	6	0	6	cervix	Age
GSE6004	4	4	0	thyroid	Gender; Age
GSE6283	6	6	0	chorion_villus_cells	tissue;Age;gender;karyotype
GSE6532	80	0	80	breast	
GSE6565	10	10	0	fetal_cartilage	
GSE6573	2	2	0	placenta;adipose_tissue	
GSE6764	23	1	22	liver	
GSE6791	22	4	18	cervix	Case;Anatomical sites;gender;age;Tumor Stage
GSE7224	9	9	0	gingival_epithelium;tonsil_epithelium	
GSE7305	10	10	0	endometrium	Menstrual phase - Follicular;Menstrual phase - Luteal
GSE7307	69	69	0	ovary;breast;synovial_membrane;penis;colon;cerebellum;kidney;prostate_gland;placenta;salivary_gland;skeletal_muscle;testes;stomach;trachea;skin;thyroid;cecum;adipose_tissue;endometrium;myometrium;mammary_gland;hippocampus;urethra;deltoid_muscle	Tissue/Cell Line [C];Disease/Normal or Treatment [C];Gender;Disease type
GSE7508	3	3	0	jurkat_cells	wild-type Jurkat cells;wild type Jurkat cells
GSE7553	22	4	18	skin	tumor size
GSE7562	2	2	0	a431_cells	
GSE7586	10	10	0	placenta	Parity;StudyID;Inflammation;ChronicPM;PM status;%parasitemia;InfantSex;MaternalAge;Birthweight(kg)
GSE7696	70	0	70	brain	patient; age; gender; treatment; survival status; survival time in months
GSE7753	25	25	0	pbmc	

Supplementary Table 1. Curated dataset of Affymetrix HGU133plus2 microarrays.

GSE7832	5	5	0	airway_epithelial_cells	Age;Sex;Ethnic group;Smoking status
GSE7832;GSE8545	1	1	0	airway_epithelial_cells	Age;Sex;Ethnic group;Smoking status
GSE7835	3	3	0	u251_cells	
GSE7904	1	0	1	breast	
GSE8050	2	2	0	kidney	age;gender;tissue mass
GSE8671	30	7	23	rectum_mucosa;sigmoid_colon_mucosa	Tissue;Location;Size;Patient ID;CEL Filename
GSE8977	10	6	4	breast_stroma	
GSE9089;GSE9196	5	5	0	cd31+_cells	
GSE9090;GSE9196	5	5	0	cd49a+_cells	
GSE9171;GSE9200	13	0	13	glioblastoma	
GSE9195	68	0	68	breast	samplename;series;age;grade;size;er;pgr;node;t.rfs;e.rfs;t.dmfs;e.dmfs;treatment
GSE9254	10	10	0	sigmoid_colon;rectum;cecum	
GSE9438	31	0	31	brain	ER;PR
GSE9489;GSE9493	13	13	0	kidney	
GSE9599	35	0	35	pancreas	
GSE9624	6	6	0	omental_adipose_tissue	Tissue;Group;Gender;Age;z score BMI
GSE9829;GSE9843	48	0	48	liver	BCLC_staging ;Age ;Sex ;Code ;Class ;AFP ;TP53 mutation ;CTNNB1 mutation ;Immunohistochemistry
GSE9834	3	3	0	astrocytes	Cell strain;Passage;Growth Medium; Insulin, 1.25 ml; Ascorbic Acid, 0.5 ml; GA-1000, 0.5 ml, L-Glutamine, 5.0 ml; FBS, 15 ml.;Grown at 37C in 5%CO2, seeded at 5000 cells/cm2;Untreated
GSE9891;GSE9899	221	0	221	ovary;peritoneum;fallopian_tube	Primary.Site ;Type ;Subtype ;StageCode ;Consolidated.Grade
GSE9984	12	12	0	placenta	first trimester;second trimester;term placenta
total	4826	688	4138		

Supplementary Table 2. Gene ontology category enrichment analysis of hyper-variable genes in cancer.

Gene ontology ID	Odds ratio	Expected count	Observed count	Category size	Adjusted P-value	Gene ontology term	Gene symbols
GO:0007275	1.80	99.44	155	1873	7.067E-06	multicellular organismal development	NOX1;COL10A1;IBSP;SPRR1B;DCN;DCN;SOX11;SPRR1A;FN1;PCDHB2;ONECUT2;SFTPB;SOX11;SOX11;ONECUT2;CSPG5;FGF20;DCN;LPPR1;HES6;HMGA2;CXCL17;CTNND2;ODAM;HMGA2;EN2;MYT1;PCDHB3;COL8A1;ARX;MYT1L;HMGA2;S100A7;FGF14;LUM;SCN2A;DKK4;PCDHB6;C14orf39;CHL1;GABRA5;DCN;PLEKHB1;FN1;TNFRSF17;FGF14;KRT14;CTNND2;GRIN2A;NEFH;SLC1A3;SH3GL3;LY6H;CACNA1D;FGFR3;IGFBP5;PAX3;ITGB6;REG3A;GPR98;LY6D;UGT8;NGFRAP1;SHISA2;KRT5;COL3A1;SPRR1A;FREM2;FAM5C;BNC1;SEMA6A;ACSL6;ARSE;GART;TLL2;USH2A;C1S;FGF14;HTN3;COL10A1;INA;COL3A1;COL15A1;CHRM3;SULF1;GRIN2A;COL3A1;COL6A1;FHL1;PKP1;SLC1A3;NDN;KLK7;DOK5;SOHLH2;PROM1;KIF5C;KLK6;MST1R;SERPINB5;PCDHB5;LEFTY1;PTPN22;PCDHB16;KRT17;IGSF9;NHLH2;INSM1;COL6A3;NRTN;CALML5;ERBB3;ERBB3;OLFM3;COMP;KRT15;COL12A1;SOX3;PTN;TFAP2B;MMP2;COL1A2;COL9A3;SLITRK5;ERBB4;ASS1;ENAM;SEMA6A;FN1;NOX1;KLK5;ELAVL3;COL5A1;POSTN;JPH1;FN1;CELSR3;FN1;SFTPB;CBS;MPPED2;CRB1;SLITRK6;DDX25;PDZRN3;CD3D;APP;CBS;CHRNA1;TIMP3;FN1;ECE2;PTPN22;E2F5;CSTA;SULF1;MPPED2;TYR;OBSL1;SIX6;TFAP2B;SHOX2;OPCML;SULF2;COL5A1;NTM;NTM;CHL1;POSTN;L1CAM;PCSK9;OLFM3;NPNT;ITGB6;FUT9;LRP2;PTN;OBSL1;DCX;MAEL;DCX;UNC5A;NR2F1;KRT17;GPR65;FN1;SULF1;MYT1L;PTPRZ1;FMOD;CD72;GHR;DYX1C1;PCDHB10;VAV3;NDP;FGF13;NEFH;EPHB1;KLK7;VAV3;ANO1

GO:0007155	2.66	21.29	50	387	1.752E-05	cell adhesion	COL11A1;COL11A1;IBSP;COL11A1;FN1;LGALS4;MUC4; MUC5B;MUC16;MSLN;CLCA2;MUC4;CHL1;FN1;ITGB6;FERMT1;LY6D;CLCA2;MUC4;COL3A1;MEGF10;CLCA2;CXCL12;FERMT1;COL3A1;COL15A1;COL3A1;COL6A1;PKP1;CCL11;MUC5AC;SLAMF7;COL6A3;TPBG;COMP;COL12A1;FN1;MTSS1;COL5A1;POSTN;FN1;FN1;THBS2;CLCA2;APP;SPON1;FN1;ITGBL1;HOXD3;FLRT3;OPCML;COL5A1;NTM;NTM;CHL1;POSTN;SELL;NPNT;ITGB6;PERP;NID2;EFS;WISP1;TRIP6;FLRT3;ANTXR1;ISLR;FN1;AZGP1;CLEC4A;CD72;MYBPC2
GO:0051674	2.24	33.48	67	588	2.937E-05	localization of cell	NOX1;NKX2-1;MMP1;FN1;ONECUT2;ONECUT2; DNAH5;S100A2;ARX;S100A7;ASCL1;SIX1;POU4F1;FOXD1;FN1;ASCL1;FOXE1;FAP;IGFBP5;GJA1;CALCA;LAMA3;FERMT1;SEMA3A;PTPRM;FOXD3;CXCL12;FERMT1;TGFB2;CDH2;NDN;POU3F2;CCL11;COL1A1;NRTN;TREM1;CALCA;PDGFRA;NKX2-3;MSN;COL1A2;ERBB4;NKX2-1;FN1;NOX1;COL5A1;MMP12;CAV1;FN1;SEMA3A;FN1;RARRES2;SFRP1;FN1;TNFSF11;CALCA;BAMBI;GAS6;IL8;SERPINE2;SRPX2;COL5A1;COL1A1;L1CAM;CTHRC1;VIL1;SELL;RASGEF1A;ASTN1;POU4F1;SFRP2;SST;TRIP6;NR2F1;FN1;CD48;PTPRM;S100A14;DYX1C1;VAV3;OLR1;PRSS3;VAV3;LBP;ENPP2;ARAP3
GO:0009887	4.52	5.65	21	103	8.886E-05	organ morphogenesis	NKX2-1;DCN;CALB1;DCN;CALB1;ONECUT2;SFTPB; ONECUT2;DCN;DCN;LY6H;PAX3;PAX6;SEMA6A;FOXA2;PAX6;FHL1;SYCP2; WT1;COMP;SOX3;NKX2-1;SEMA6A;SFTPB;PITX2;E2F5;SIX6;PBX1;NOTCH2;IRX2
GO:0040011	2.01	37.83	69	675	3.806E-04	locomotion	NOX1;CXCL11;CXCL11;FOXG1;MMP1;FN1;DNAH5; CXCL17;CCL18;S100A2;CCL18;ARX;ASCL1;ETV4;CXCL6;JPH3;CHL1;SIX1;POU4F1;FOXD1;FN1;ASCL1;FOXE1;FAP;CACNA1D;CXCL9;GJA1;CALCA;FERMT1;BMP7;COL3A1;FOXD3;SEMA6A;FERMT1;COL3A1;COL3A1;COL6A1;CDH2;NDN;POU3F2;KIF5C;COL6A3;NRTN;TREM1;CALCA;NKX2-3;MSN;COL1A2;COL9A3;SEMA6A;FN1;NOX1;LHX2;COL5A1;MMP12;CAV1;FN1;FN1;APP;FN1;TNFSF11;CALCA;BAMBI;GAS6;COL5A1;CHL1;L1CAM; ATP2B2;CCL28;CTHRC1;SELL;RASGEF1A;ASTN1;POU4F1;DCX;CXCL5;DCX; UNC5A;NR2F1;HOXA1;SERPIND1;FN1;CD48;CD72;DYX1C1;VAV3;OLR1;PRSS3;EPHB1;VAV3

GO:0030199	9.86	1.54	10	27	4.918E-04	collagen fibril organization	COL11A1;COL11A1;COL11A1;LUM;COL3A1;COL3A1; TGFB2;COL3A1;COL1A1;COL12A1;COL1A2;LOX;COL5A1;COL5A1;COL1A1; LOX;SFRP2
GO:0071842	4.44	4.65	17	82	9.589E-04	cellular component organization at cellular level	NOX1;COL11A1;COL11A1;IBSP;COL11A1;TNFRSF11B; LUM;TGFB2;TNFRSF11B;SERPINB5;MUC5AC;WT1;PDGFRA;COL12A1;ERB B4;NOX1;POSTN;PDZRN3;CHRNA1;POSTN;SFRP2;ABI3BP
GO:0006928	2.16	23.34	46	416	2.467E-03	cellular component movement	NOX1;MMP1;FN1;DNAH5;S100A2;ARX;ASCL1;SIX1; POU4F1;FOXD1;FN1;ASCL1;FOXE1;FAP;DNALI1;GJA1;CALCA;FERMT1;FO XD3;FERMT1;CDH2;NDN;POU3F2;MST1R;SERPINB5;NRTN;TREM1;CALCA ;NKX2- 3;MSN;COL1A2;FN1;NOX1;MTSS1;COL5A1;MMP12;CAV1;FN1;FN1;FN1;T NFSF11;CALCA;BAMBI;GAS6;COL5A1;L1CAM;CTHRC1;SELL;RASGEF1A;AS TN1;POU4F1;NR2F1;FN1;CD48;DNALI1;DYX1C1;VAV3;OLR1;PRSS3;VAV3; DNAH9
GO:0007156	3.34	6.89	20	121	3.585E-03	homophilic cell adhesion	PCDHB2;DSG3;PCDHB3;DSC3;PCDHB6;CDH6;DSG3 ;FREM2;PTPRM;CDH6;DSC3;CDH2;PCDH8;PCDHB5;PCDHB7;PCDH19;PCD HB16;PCDH7;CELSR3;FAT1;L1CAM;PCDHA9;PTPRM;CDH6;PCDHB10
GO:0009954	7.93	1.59	9	28	3.585E-03	proximal/distal pattern formation	DLX2;DLX1;EN1;TP63;HOXC10;HOXA9;SP8;PBX1;IRX2
GO:0007267	2.52	13.64	31	245	3.585E-03	cell-cell signaling	CXCL11;SIX3;CXCL11;SOX2;PTHLH;NKX2-1;PTHLH; WISP3;TSHR;FOXA1;TSHR;FGF20;PTHLH;CCL18;CCL18;FGF14;CXCL6;FOX A1;SIX1;CEACAM6;FGF14;CEACAM6;CXCL9;GJA1;CALCA;SH2D1A;FGF14; TGFB2;PCDH8;STC2;CALCA;NKX2- 1;CRB1;FAT1;ECE2;SOX2;CALCA;AR;SH3KBP1;SFRP2;CXCL5;WISP1;TSHR; NDP;FGF13
GO:0050910	27.78	0.46	5	8	5.995E-03	detection of mechanical stimulus involved in sensory perception of sound	COL11A1;COL11A1;COL11A1;SOX2; GPR98;SOX2;CHRNA9;ATP2B2

GO:0022008	1.92	30.60	54	552	6.170E-03	neurogenesis	LHX8;ONECUT2;ONECUT2;FGF20;PCSK1;EN2;ARX; ETV4;EN1;CHL1;GRIN2A;SLC1A3;CACNA1D;FGFR3;GJA1;GPR98;COL3A1; PTPRM;SEMA6A;ACSL6;USH2A;COL3A1;GRIN2A;COL3A1;COL6A1;SLC1A3;PROM1;KIF5C;COL1A1;IGSF9;PAX7;COL6A3;NRTN;HOXC10;OLFM3;COL1A2;COL9A3;SLITRK5;SEMA6A;LHX2;COL5A1;CELSR3;CRB1;SLITRK6;APP;OPCML;FZD8;COL5A1;COL1A1;NTM;NTM;CHL1;L1CAM;ATP2B2;PCSK9;CTHRC1;OLFM3;DCX;DCX;UNC5A;NR2F1;HOXA1;FZD8;PTPRM;PTPRZ1;CD72;DYX1C1;EPHB1
GO:0006958	6.84	1.77	9	31	6.975E-03	complement activation, classical pathway	IGKV4-1;IGKC;C1S;MBL2;CRP;C4BPB;C7;C2;CFI
GO:0016339	7.87	1.42	8	25	8.102E-03	calcium-dependent cell-cell adhesion	PCDHB2;PCDHB3;PCDHB6;CDH2;PCDHB5;PCDHB7; PCDHB16;PCDHB10
GO:0002253	5.58	2.28	10	40	9.482E-03	activation of immune response	IGKV4-1;IGKC;CFHR5;C1S;MBL2;CRP;C4BPB;C7;C2;CFI
GO:0045665	6.02	1.94	9	34	1.281E-02	negative regulation of neuron differentiation	SOX2;FOXG1;NKX2-2;ASCL1;ASCL1;BMP7;FOXA2;ID4; SOX2;PBX1;ASPM
GO:0048706	8.41	1.19	7	21	1.418E-02	embryonic skeletal system development	DLX1;DLK1;COL1A1;PAX7;HOXA9;COL1A1; HOXD1;PBX1
GO:0050982	11.12	0.85	6	15	1.418E-02	detection of mechanical stimulus	COL11A1;COL11A1;COL11A1;SOX2;GPR98;TRPA1;SOX2;CHRNA9;ATP2B2
GO:0072376	5.07	2.45	10	43	1.420E-02	protein activation cascade	IGKV4-1;IGKC;CFHR5;C1S;MBL2;CRP;C4BPB;C7;C2;CFI
GO:0001501	3.44	5.01	15	90	1.420E-02	skeletal system development	COL10A1;TNFRSF11B;EN1;COL3A1;DLL3;ARSE;COL10A1;COL3A1;COL3A1; TNFRSF11B;TP63;HOXC10;COMP;COL12A1;TEAD4;COL1A2;POSTN;SHOX2;POSTN;ANKH

GO:0009605	1.65	48.13	74	856	1.420E-02	response to external stimulus	CXCL11;CXCL11;NKX2-1;FOXG1;PCSK1;CXCL17;CCL18;CCL18;ARX;S100A7; TNFRSF11B;MMP3;ASCL1;ETV4;CXCL6;CHL1;FOXD1;PLEKHB1;ASCL1;CACNA1D;CXCL9;CALCA;BMP7;COL3A1;SDR16C5;PTPRM;FOXD3;SEMA6A;F12;TNFSF4;CXCL12;COL3A1;TGFB2;FOXA2;COL3A1;COL6A1;TNFRSF11B;CPB2;TSPAN8;CCL11;KIF5C;COL6A3;STC2;NRTN;CALCA;INHBB;COL1A2;COL9A3;CNR1;NKX2-1;ASS1;SEMA6A;LHX2;COL5A1;CBS;RARRES2;SCGB1A1;APP;SFRP1;CBS;SERPINA7;TNFSF11;CALCA;IL8;SERPINE2;COL5A1;CHL1;L1CAM;CCL28;PCSK9;HRK;LRP2;SFRP2;DCX;SST;CXCL5;DCX;UNC5A;HOXA1;SERPIND1;PTPRM;CD72;RBP1;S100A14;GHR;EPHB1;LBP;ENPP2;CCNE1
GO:0007586	4.50	2.96	11	52	1.439E-02	digestion	AKR1B10;CTSE;TFF3;CHRM3;AKR1C2;TFF2;MUC5AC;PRSS2;AKR1C2;SST; CYP39A1;PRSS3
GO:0030574	7.79	1.25	7	22	1.585E-02	collagen catabolic process	MMP1;MMP13;MMP3;KLK6;MMP2;PRSS2;MMP7
GO:0048856	2.10	17.60	34	359	1.722E-02	anatomical structure development	COL10A1;PCDHB2;CSPG5;LPPR1;HES6;MYT1;PCDHB3;MYT1L;FGF14;SCN2A; PCDHB6;FGF14;NEFH;SH3GL3;UGT8;DLK1;FAM5C;ACSL6;ARSE;C1S;FGF14;COL10A1;CHRM3;ARNT2;DOK5;PCDHB5;PCDHB16;NHLH2;COL12A1;ELAVL3;MPPED2;CD40;MPPED2;HOXD1;FUT9;MYT1L;DYX1C1;PCDHB10;FGF13;NEFH
GO:0022612	6.12	1.70	8	30	1.874E-02	gland morphogenesis	CDKN2A;CDKN2A;PTHLH;PTHLH;PTHLH;ETV4;SFRP4;CCL11;ERBB3;ERBB3; NKX2-3;CAV1;SFRP4
GO:0044259	4.52	2.68	10	47	2.377E-02	multicellular organismal macromolecule metabolic process	MMP1;MMP13;MMP3;COL3A1;COL3A1;COL3A1;KLK6;COL1A1;MMP2;PRSS2; COL5A1;MMP7;COL5A1;COL1A1
GO:0016540	22.19	0.40	4	7	2.795E-02	protein autoprocessing	PCSK1;F12;KLK6;PCSK9
GO:0032355	3.46	4.32	13	76	2.795E-02	response to estradiol stimulus	TFF1;FOXA1;MMP3;FOXA1;BMP7;SFRP4;ARNT2;CCND2;PDGFRA;ASS1;SFRP4; SLC6A1;GHR;SOCS2;CCNE1

Supplementary Table 5: Cancer-specific analysis of tissue specificity and hyper-variability. We computed the proportion of hyper-variable genes that are specific to each of the seven tissues with sufficient samples of both normal and cancer. The vast majority of hyper-variable genes are not tissue-specific in these tissues (column 2). However, there is enrichment of hyper-variability in tissue-specific genes (columns 3 and 4). We repeated the same analysis for differentially expressed genes, but found no enrichment in the set of tissue-specific genes.

	Number of hyper-variable genes	Pct. that are tissue-specific	OR	P-value
**adrenal cortex**	14293	5.11	5.02	1.39E-156
**colon**	8882	3.25	4.23	5.89E-65
**endometrium**	5808	1.05	2.08	1.84E-06
**kidney**	4290	3.19	5.73	3.48E-48
**skin**	9590	1.89	2.96	7.04E-27
**stomach**	4906	1.35	2.39	7.67E-09
**vulva**	5323	3.57	5.33	5.19E-60

	Number of down-regulated genes	Pct. that are tissue-specific	OR	P-value
**adrenal cortex**	1790	2.46	1.17	3.16E-01
**colon**	4218	0.97	0.80	1.84E-01
**endometrium**	6560	0.55	0.97	9.30E-01
**kidney**	4826	0.64	0.81	3.03E-01
**skin**	7754	0.67	0.75	4.68E-02
**stomach**	2427	0.45	0.70	2.96E-01
**vulva**	5221	0.84	0.86	3.73E-01

	Number of up-regulated genes	Pct. that are tissue-specific	OR	P-value
**adrenal cortex**	2543	1.93	0.90	5.26E-01
**colon**	7558	0.98	0.80	7.62E-02
**endometrium**	5437	0.59	1.05	7.75E-01
**kidney**	6509	0.66	0.83	2.92E-01
**skin**	8625	0.93	1.09	4.86E-01
**stomach**	5118	0.47	0.72	1.17E-01
**vulva**	4687	1.11	1.16	3.11E-01

Supplementary Table 6. Anti-profile signatures are not explained by pathological tumor stage or grade heterogeneity. For each of the leave-one-tissue-out experiments reported in Figure 4, we used available annotated pathological tumor stage or grade to find genes that are differentially expressed across clinical stages or grades. The first column in the table indicates the number and percentage of genes that show significant pathological stage or grade differences (FDR<0.1). The second column indicates the number and percentage of these genes included in the 100-gene anti-profile used to classify samples in the leave-one-tissue-out experiment. The last two columns are odds ratio and P-value for a Fisher exact test for enrichment of differentially expressed genes in the anti-profile. For adrenal cortex, stomach and vulva there are very few differentially expressed genes (22, 2 and 4 respectively).

	**Number of differentially expressed genes**	**Number in anti-profile**	**OR**	**P-value**
**adrenal cortex**	22 (0.040%)	1 (4.545%)	26.2	0.040
**colon**	9271 (16.976%)	29 (0.313%)	2.0	0.003
**endometrium**	4377 (8.015%)	15 (0.343%)	2.0	0.016
**kidney**	8183 (14.984%)	13 (0.159%)	0.8	0.675
**skin**	11482 (21.024%)	22 (0.192%)	1.1	0.806
**stomach**	2 (0.004%)	0 (0.000%)	0.0	1.000
**vulva**	4 (0.007%)	0 (0.000%)	0.0	1.000

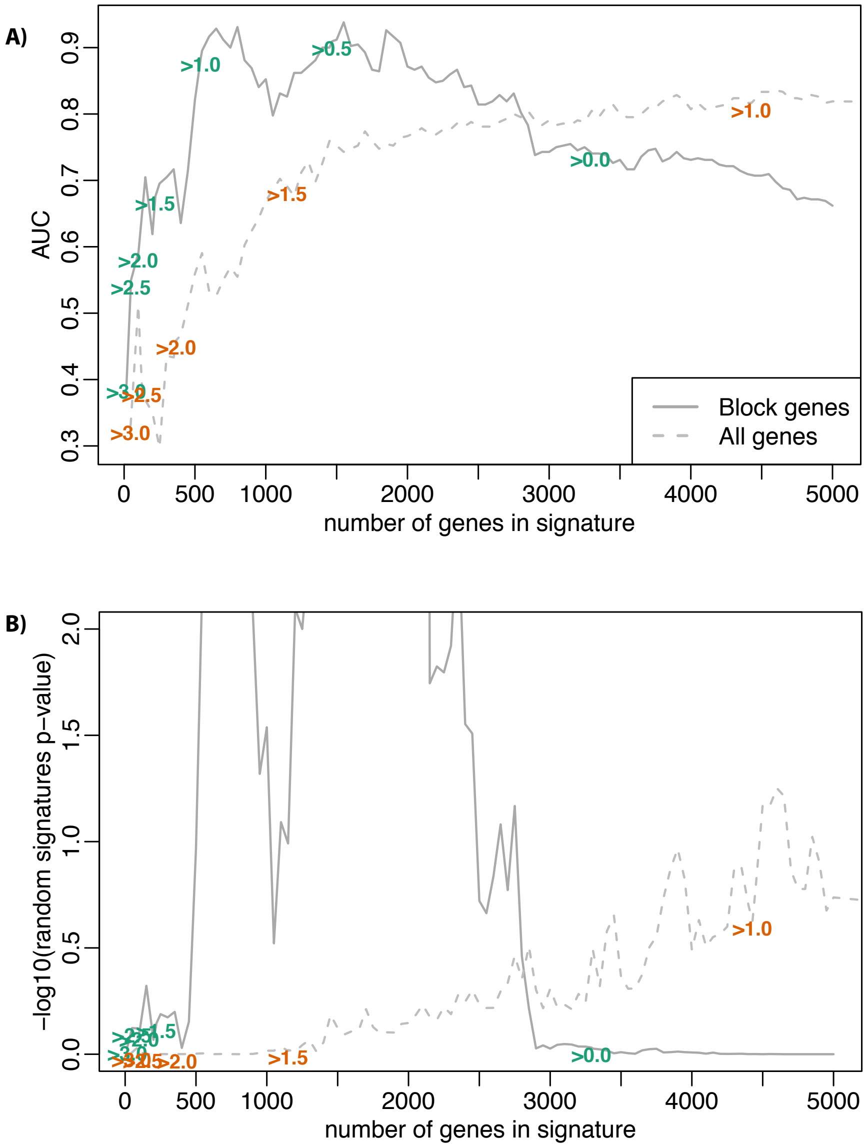

Supplementary Figure 1. Sensitivity and significance of colon cancer peripheral blood signature. (A) AUCs obtained by signatures of varying size. The solid line corresponds to signatures built using only genes inside blocks of cancer methylation changes [23], while the dashed line corresponds to all genes. Cutoffs for the log2 variance ratio statistic are indicated along the curve. The signature reported in the manuscript includes genes in blocks (solid line) with cancer variability at least twice that of normal (>1) which obtained AUC of 0.89. Similar AUCs are obtained with signatures with about 500-2000 genes inside blocks. This highlights the importance of including genes inside blocks of methylation changes in colon cancer. (B) The proportion of random signatures of corresponding size obtaining an AUC greater than or equal to the anti-profile. Results show that significantly high AUC was obtained by signatures that include about 500-2000 of the top hyper-variable genes inside methylation blocks and therefore the results discussed in the main text are not very sensitive to signature size. For signatures greater than 3000 genes, the anti-profiles built from all genes perform better than anti-profiles built from only genes inside blocks of cancer methylation changes. This is due to the fact that while hyper-variable genes are enriched in these blocks 2,047 of the 5,339 genes inside these blocks are not hyper-variably expressed, and their inclusion degrades anti-profile performance.

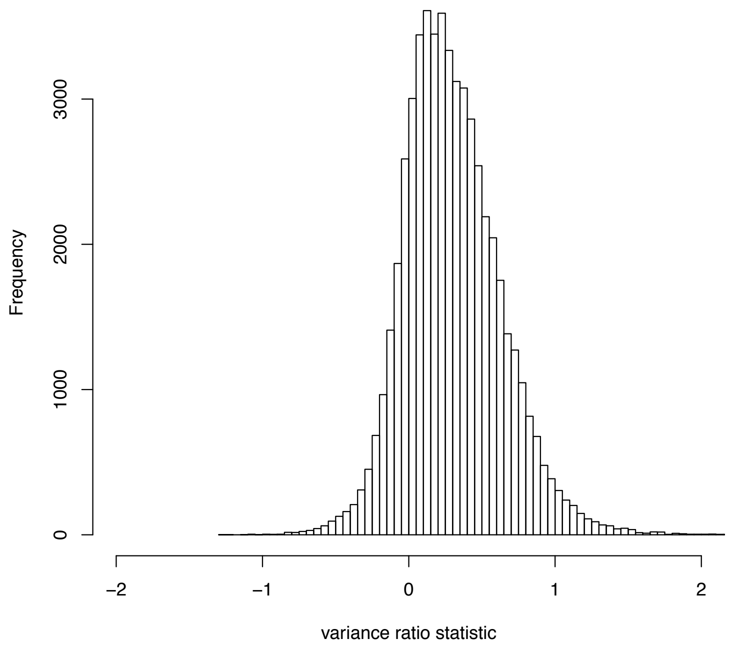

Supplementary Figure 2. Histogram of log2 cancer/normal variance ratio. We computed a statistic to determine general hyper-variability in cancer as discussed in Methods and Materials. Here we plot the histogram of this statistic across the ~55,000 probesets in the HGU133plus2 microarray. While a majority of probes show increased variability in cancer (log2 ratio >0), relatively few genes (1,456) show hyper-variability (log2 ratio >1).

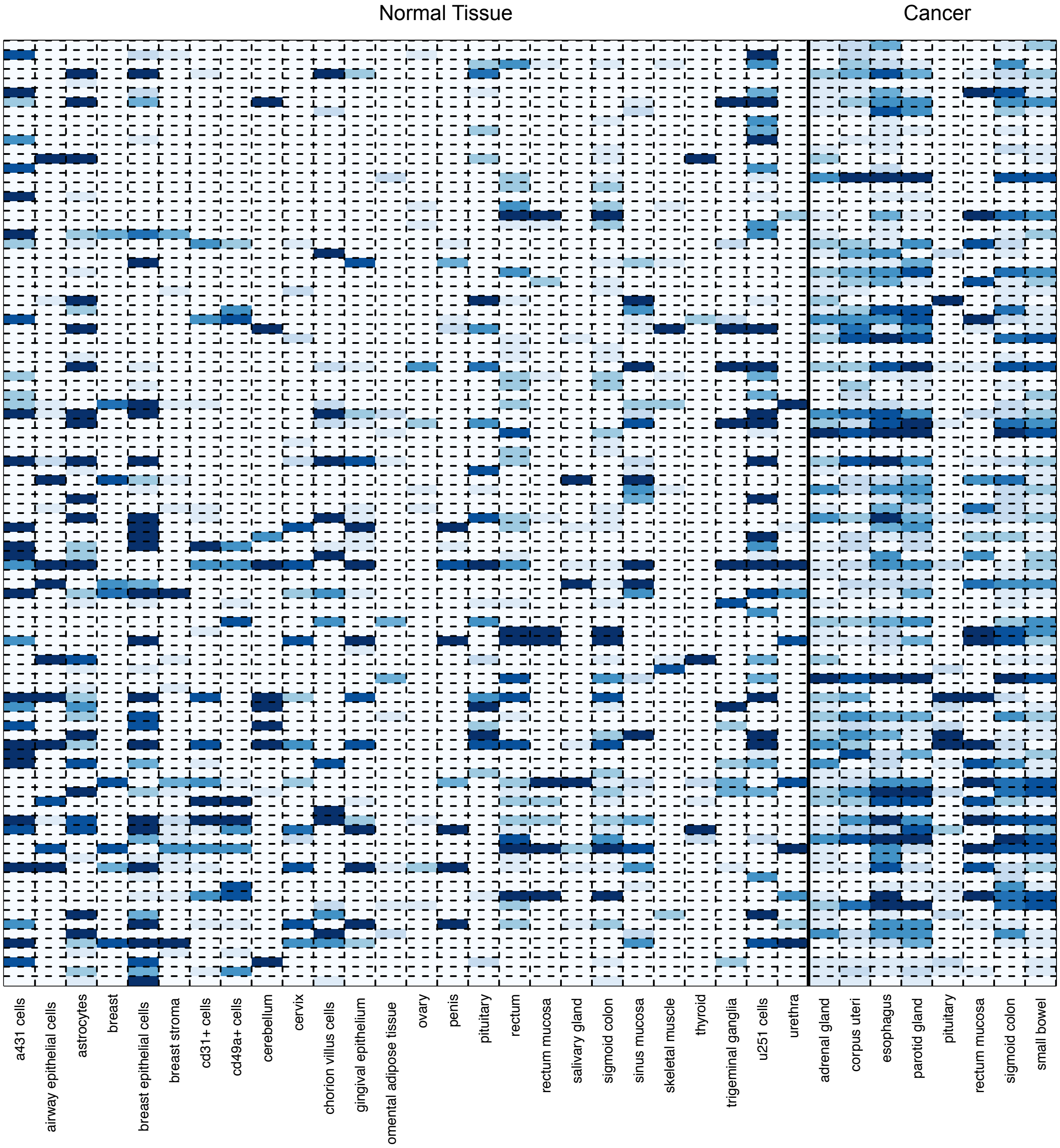

Supplementary Figure 3. Genes with consistent hyper-variability across cancer types. A plot of the 100 genes that most consistently show hyper variability across cancer types, similar to Figure 3A, on tissue types and cancers not used to define hyper-variability. As for the samples in Figure 3A, we observed that for the majority of genes, the percentage of samples in each normal tissue type outside normal range is close to either 0% (most tissues) or 100% (the small number of tissues for which the gene is tissue-specific). We also observe that in cancer, percentages are consistently away from 0% or 100%, indicating high variability.

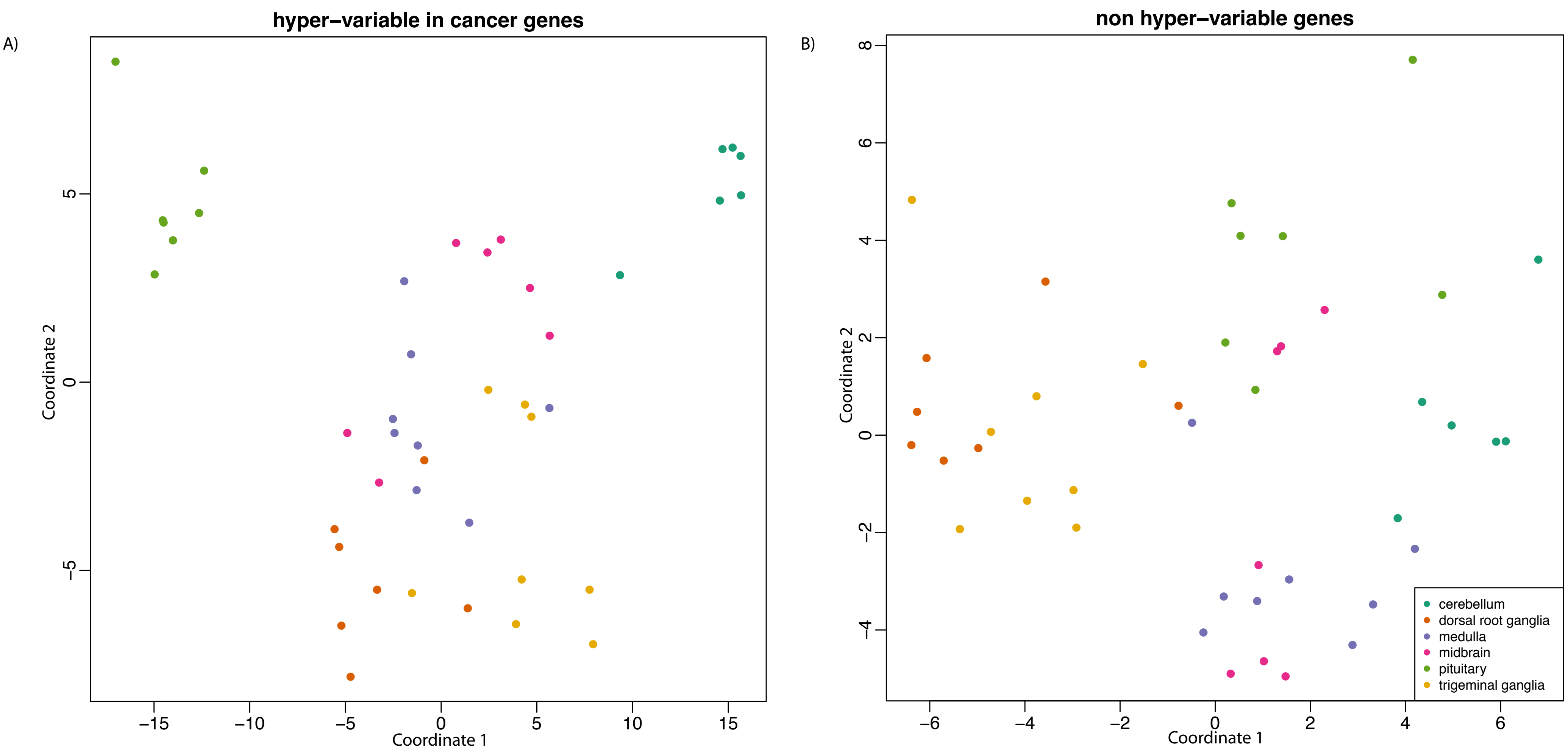

Supplementary Figure 4. Hyper-variable genes in cancer distinguish normal samples from different brain regions. (A) Multi-dimensional scaling of gene expression of hyper-variable genes in cancer from normal samples of different brain regions. (B) Multi-dimensional scaling of gene expression in 100 randomly selected non-hyper-variable genes in cancer for the same samples of different brain regions. For genes that are hyper-variable in cancer, samples cluster by brain region indicating their tissue specificity. However, the set of control genes (non-hyper-variable in cancer), lack tissue specificity and samples do not cluster by brain region. We computed ratios of between-cluster variance to within-cluster variance for both hyper-variable and control and observed that brain region clusters are tighter for hyper-variable genes than control genes (variance ratios of 60.5 and 4.2 for hyper-variable and control genes respectively).

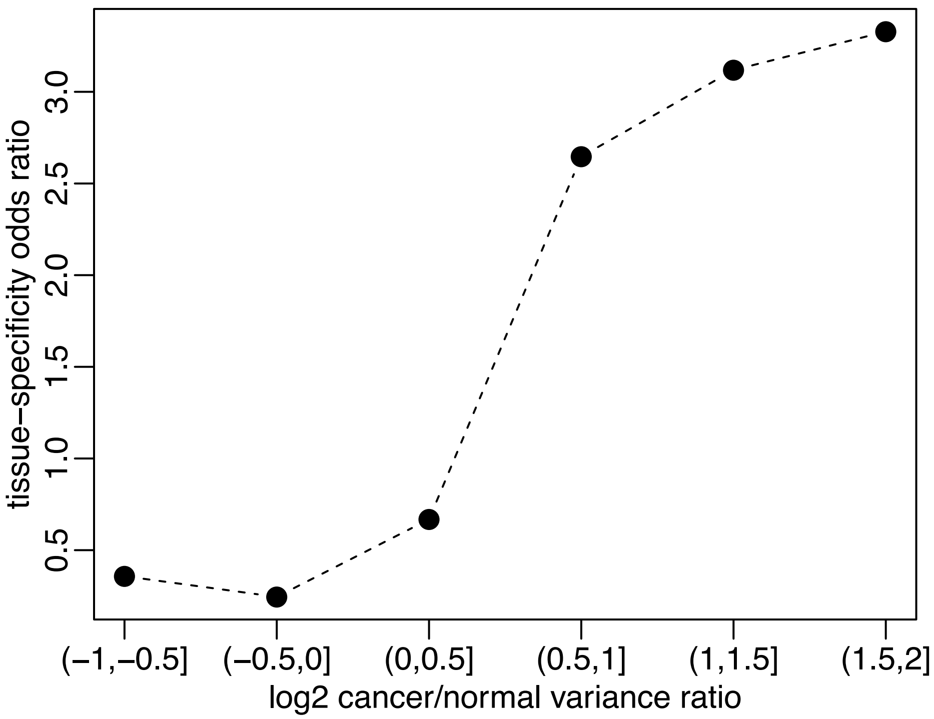

Supplementary Figure 5. The set of tissue-specific genes is enriched for genes that are hyper-variable in cancer. We plot the odds ratio for a hyper-variability enrichment Fisher exact test in tissue-specific genes as a function of the log2 variance ratio. Each point corresponds to genes with log2 variance ratio within the indicated range. Odds ratio increases as variability in cancer increases. All tests are significant at $P<0.05$ level.

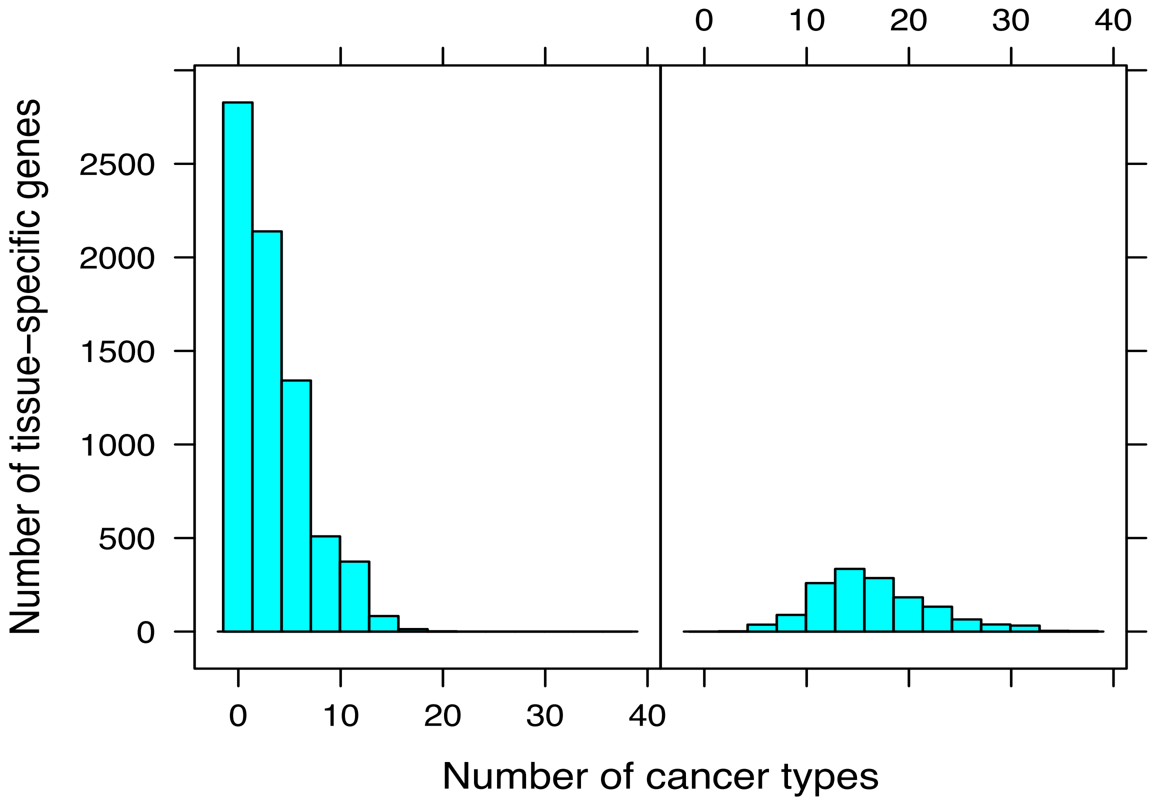

Supplementary Figure 6. Specificity of the universally hyper-variable tissue-specific genes. For each tissue-specific gene, we calculate the number of cancer types for which it is hyper-variable by computing the log2 ratio between standard deviation for that cancer type and mean normal standard deviation across tissues. We plot on the left a histogram of the number of cancer types for tissue-specific genes that are not consistently hyper-variable in cancer. The histogram on the right corresponds to genes that are consistently hyper-variable. We see that the vast majority of tissue-specific genes show hyper-variability in a small number of cancer-types. This suggests that consistent hyper-variability in cancer cannot be ascribed entirely to cellular heterogeneity.

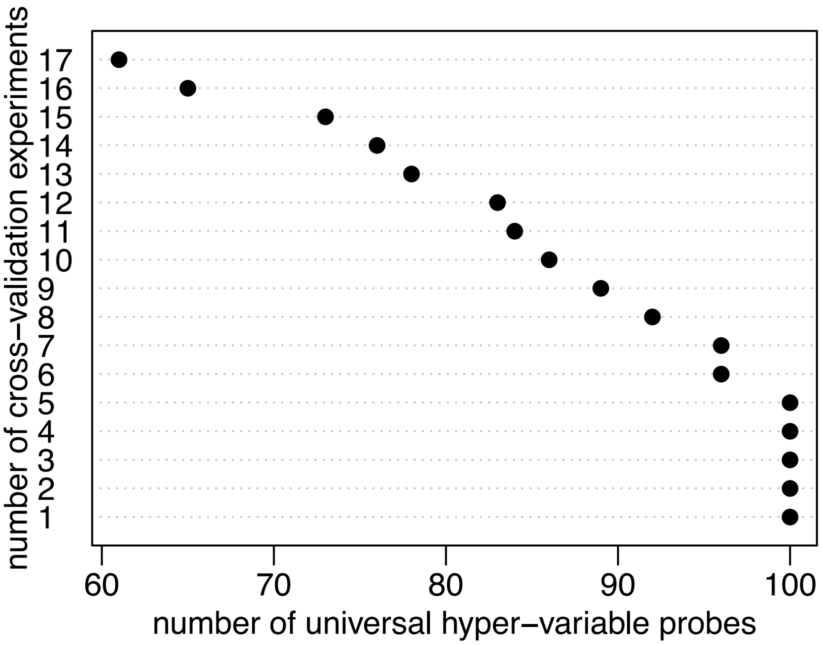

Supplementary Figure 7. The anti-profile method produces robust signatures. We plot the percentage of the top 100 universally hyper-variable genes selected in at least *n* cross-validation experiments. We observed that more than 73% of the universally hyper-variable genes are selected in 15 or more of the 17 cross-validation experiments performed, while all of the hyper-variable genes were selected in at least 5 cross-validation experiments, indicating the robustness and reproducibility of the anti-profile method.

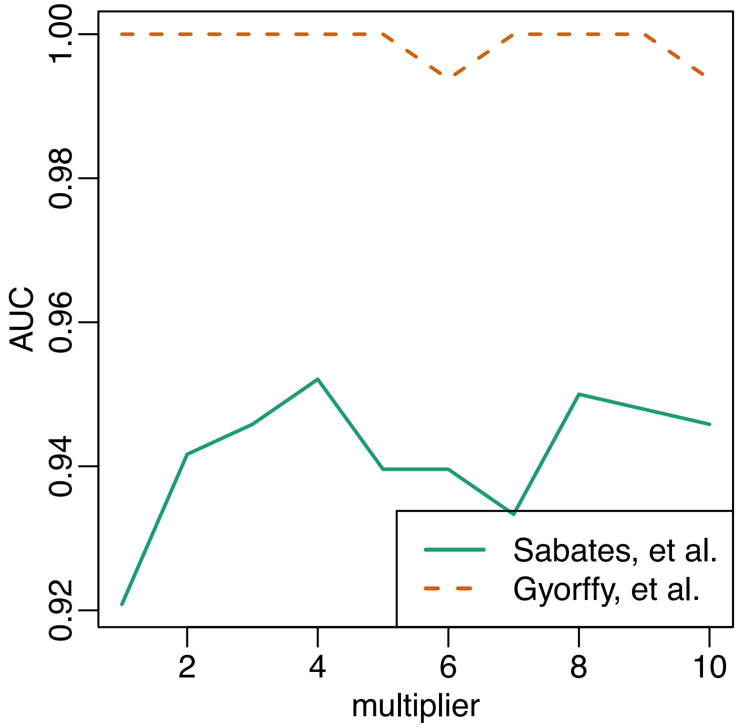

Supplementary Figure 8. Sensitivity of colon anti-profile to choice of normal expression region multiplier. We repeated the cross-validation experiment reported in Figure 1B using different median absolute deviation multipliers to define normal regions of expression. Here we plot the AUC achieved in cross-validation against the choice of multiplier. We find that results are not very sensitive to the choice of multiplier except for narrow regions of normal expression (1-3) where performance drops in one of the datasets.
